# Invasive Treatment Strategy in Adults With Frailty and Non–ST-Segment Elevation Myocardial Infarction: A Secondary Analysis of a Randomized Clinical Trial

**DOI:** 10.1001/jamanetworkopen.2024.0809

**Published:** 2024-03-06

**Authors:** Juan Sanchis, Héctor Bueno, Sergio García-Blas, Oriol Alegre, David Martí, Manuel Martínez-Sellés, Laura Domínguez-Pérez, Pablo Díez-Villanueva, Jose A. Barrabés, Francisco Marín, Adolfo Villa, Marcelo Sanmartín, Cinta Llibre, Alessandro Sionís, Antoni Carol, Agustín Fernández-Cisnal, Elena Calvo, María José Morales, Jaime Elízaga, Iván Gómez, Fernando Alfonso, Bruno García del Blanco, Francesc Formiga, Eduardo Núñez, Julio Núñez, Albert Ariza-Solé

**Affiliations:** 1 Cardiology Department, University Clinic Hospital of València, University of València, Instituto de Investigación Sanitaria Clínico Valencia, Centro de Investigación Biomédica en Red Enfermedades Cardiovaculares (CIBERCV), Valencia, Spain; 2 Centro Nacional de Investigaciones Cardiovasculares, Madrid, Spain; 3 Cardiology Department, Universisty Hospital 12 de Octubre and Instituto de Investigación Sanitaria Hospital 12 de Octubre (imas12), CIBERCV, Madrid, Spain; 4 Complutense University, Madrid, Spain; 5 Cardiology Department, University Hospital of Bellvitge, L’Hospitalet de Llobregat, Barcelona, Spain; 6 Central Defense Hospital, Alcalá University, Madrid, Spain; 7 Cardiology Department, University Hospital Gregorio Marañón, CIBERCV, Complutense University, European University, Madrid, Spain; 8 University Hospital La Princesa, Autonomous University of Madrid, Instituto de Investigación Sanitaria Princesa, CIBERCV, Madrid, Spain; 9 University Hospital Vall d’Hebron, CIBERCV, Barcelona, Spain; 10 University Hospital Virgen de la Arrixaca, Instituto Murciano de Investigación Biosanitaria–Arrixaca, CIBERCV, El Palmar, Murcia, Spain; 11 Southeast University Hospital, Arganda del Rey, Madrid, Spain; 12 University Hospital Ramón y Cajal, CIBERCV, Madrid, Spain; 13 University Hospital Germans Trias i Pujol, CIBERCV, Badalona, Barcelona, Spain; 14 University Hospital Sant Pau, CIBERCV, Barcelona, Spain; 15 Moisés Broggi Hospital, Sant Joan Despí, Barcelona, Spain

## Abstract

**Importance:**

The MOSCA-FRAIL randomized clinical trial compared invasive and conservative treatment strategies in patients with frailty with non–ST-segment elevation myocardial infarction (NSTEMI). It showed no differences in the number of days alive and out of the hospital at 1 year.

**Objective:**

To assess the outcomes of the MOSCA-FRAIL trial during extended follow-up.

**Design, Setting, and Participants:**

The MOSCA-FRAIL randomized clinical trial was conducted at 13 hospitals in Spain between July 7, 2017, and January 9, 2021, and included 167 adults (aged ≥70 years) with frailty (Clinical Frailty Scale score ≥4) and NSTEMI. In this preplanned secondary analysis, follow-up was extended to January 31, 2023. Data analysis was performed from April 5 to 29, 2023, using the intention-to-treat principle.

**Interventions:**

Patients were randomized to a routine invasive (coronary angiography and revascularization if feasible [n = 84]) or a conservative (medical treatment with coronary angiography only if recurrent ischemia [n = 83]) strategy.

**Main outcomes and measures:**

The primary end point was the difference in restricted mean survival time (RMST). Secondary end points included readmissions for any cause, considering recurrent readmissions.

**Results:**

Among the 167 patients included in the analysis, the mean (SD) age was 86 (5) years; 79 (47.3%) were men and 88 (52.7%) were women. A total of 93 deaths and 367 readmissions accrued. The RMST for all-cause death over the entire follow-up was 3.13 (95% CI, 2.72-3.60) years in the invasive and 3.06 (95% CI, 2.84-3.32) years in the conservative treatment groups. The RMST analysis showed inconclusive differences in survival time (invasive minus conservative difference, 28 [95% CI, −188 to 230] days). Patients under invasive treatment tended to have shorter survival in the first year (−28 [95% CI, −63 to 7] days), which improved after the first year (192 [95% CI, 90-230] days). Kaplan-Meier mortality curves intersected, displaying higher mortality to 1 year in the invasive group that shifted to a late benefit (landmark analysis hazard ratio, 0.58 [95% CI, 0.33-0.99]; *P* = .045). Early harm was more evident in the subgroup with a Clinical Frailty Scale score greater than 4. No differences were found for the secondary end points.

**Conclusions and Relevance:**

In this extended follow-up of a randomized clinical trial of patients with frailty and NSTEMI, an invasive treatment strategy did not improve outcomes at a median follow-up of 1113 (IQR, 443-1441) days. However, a differential distribution of deaths was observed, with early harm followed by later benefit. The phenomenon of depletion of susceptible patients may be responsible for this behavior.

**Trial registration:**

ClinicalTrials.gov Identifier: NCT03208153

## Key Points

**Question:**

Does a routine invasive strategy improve midterm outcomes in adults with frailty and acute non–ST-segment elevation myocardial infarction (NSTEMI)?

**Findings:**

In this secondary analysis of a randomized clinical trial of 167 patients with frailty and NSTEMI, a routine invasive strategy, when compared with a conservative strategy, did not reduce the number of days alive at a median follow-up of 1113 days. Invasive treatment was associated with shorter survival within the first year but more prolonged survival after the first year.

**Meaning:**

In patients with frailty and NSTEMI, an initial invasive strategy caused early harm followed by late benefit, resulting in a neutral effect on survival at 4 years.

## Teaser

This extended follow-up of a randomized clinical trial investigates whether restricted mean survival time differs among patients with frailty who undergo intensive vs conservative treatment for acute non–ST-segment elevation myocardial infarction.

## Introduction

Non–ST-segment elevation myocardial infarction (NSTEMI) poses significant challenges in the geriatric population, particularly among patients with frailty.^1^ While invasive cardiac procedures can provide substantial benefits, they also carry inherent risks. Moreover, the prognosis of patients with frailty is influenced by multiple factors beyond the acute coronary event.

There are limited data on the optimal treatment (invasive or conservative) of older adults with acute coronary syndrome.^2,3,4^ In the absence of robust evidence, decisions regarding how to treat older patients should be individualized based on patient characteristics. It is acknowledged that comorbidities can attenuate the potential benefit of invasive treatment.^5,6^ Clinical guidelines recommend considering ischemic and bleeding risks, estimated life expectancy, comorbidities, the need for noncardiac surgery, quality of life, frailty, cognitive and functional impairment, patient values and preferences, and the risks and benefits of an invasive strategy.^2^

To our knowledge, MOSCA-FRAIL was the first clinical trial to compare an initially invasive and a conservative treatment strategy in patients with frailty and NSTEMI. The results showed no significant differences in the number of days alive and out of the hospital at 1 year, and worse outcomes were observed among patients who underwent invasive treatment.^7^ Therefore, a conservative approach might be the best option for patients with frailty and NSTEMI. In the present study, we investigated whether these findings consolidate or change over time in the extended follow-up of the trial.

## Methods

### Study Design

The MOSCA-FRAIL study design has been described elsewhere.^7^ In brief, it was a multicenter, prospective, randomized, open-label clinical trial conducted in older adult patients with frailty and NSTEMI. The inclusion criteria consisted of (1) NSTEMI, defined by symptoms consistent with acute myocardial ischemia, absence of persistent ST-segment elevation, and troponin level elevation (according to the local laboratory troponin assay); (2) 70 years or older; and (3) frailty defined by 4 points or greater on the Clinical Frailty Scale (CFS).^8^ Participants were randomized within 48 hours of admission to 1 of the 2 treatment strategies: (1) routine invasive strategy, consisting of coronary angiography within 72 hours of admission with coronary revascularization if deemed appropriate, or (2) conservative strategy, consisting of medical therapy only, although cardiac catheterization was allowed in the case of recurrent ischemia during the index hospitalization. Medical treatment was optimized according to the clinical practice guidelines for all patients. Exclusion criteria consisted of prior known nonrevascularizable coronary artery disease, significant concomitant nonischemic heart disease, inability to understand or sign informed consent (by patients or relatives), and life expectancy of less than 12 months. In addition to the defined inclusion and exclusion criteria, the attending cardiologist believed that the participation of the patient in the study was reasonable. Reasons for considering participation inappropriate were either a recommendation by the attending cardiologist that invasive treatment be mandatory owing to severe clinical instability at admission (recurrent chest pain and/or dynamic ischemic electrocardiographic changes) or any factor that precluded invasive treatment.

The trial was an investigator-driven initiative under the auspices of the Spanish Society of Cardiology and the official working groups of Interventional Cardiology and Geriatric Cardiology. A total of 13 centers participated in the study. The recruitment period was between July 7, 2017, and January 9, 2021. The extended follow-up ended on January 31, 2023, and included all the patients enrolled in the trial (n = 167). The extended follow-up analysis was prespecified in the trial protocol (Supplement 1); the restricted mean survival time (RMST) analysis was not prespecified. All centers received the approval of their Medical Ethics Committee, and all patients provided written informed consent. This study followed the Consolidated Standards of Reporting Trials (CONSORT) reporting guideline. The study flow diagram is shown in eFigure 1 in Supplement 2.

### End Points

The original trial was designed for a primary end point of the number of days alive and out of the hospital between discharge from the index hospitalization to 1 year. With the follow-up duration extending to a median of 1113 (IQR, 443-1441) days, tracking hospitalization days became increasingly complex. Therefore, we selected the RMST differences for all-cause mortality (ie, days alive) between the treatment strategies as an alternative primary end point for this extended follow-up analysis. The RMST does not require the proportionality of the hazard over time, accounts for censored adjustment, and allows for time-dependent effect adjustment.^9^ In our RMST analysis, we modeled the time-dependent effect of the intervention strategy using restricted cubic splines with 2 *df*. This approach allows us to accurately represent the changing impact of the intervention strategy over time, providing a nuanced understanding of its effects throughout the follow-up period. Excluding the time patients spent in the hospital due to intercurrent events would have increased the complexity of the RMST analysis. The causes of death were classified as cardiac, noncardiac, and undetermined when unwitnessed or without documentation to determine the cause.

Secondary end points included the composite of all-cause death and the time to first occurrence of ischemic cardiac events (reinfarction and postdischarge revascularization), any cardiac events (reinfarction, unstable angina, coronary revascularization, acute heart failure, and other cardiac reasons), and noncardiac events (stroke, bleeding, and other noncardiac causes). Additionally, the study collected data on recurrent events. Local investigators were instructed to report and classify all events. The events were not centrally adjudicated during the extended follow-up.

### Statistical Analysis

Data analysis was performed from April 5 to 29, 2023. All statistical comparisons were made under the intention-to-treat principle. Results are presented as frequencies or mean (SD) as appropriate. Between-group comparisons were performed using the unpaired 2-tailed *t* test or Fisher exact test. We used standardized differences to evaluate how well matched the baseline characteristics resulted from the randomization of the 2 treatment groups. A standardized difference of 0.25 or less was considered a good match.

The effect of the invasive strategy on all-cause mortality was assessed using Kaplan-Meier curves. However, the proportionality assumption was violated on crossing the curves from initial harm to a late beneficial effect. Given the frailty of the study population, a plausible explanation for this bimodal effect could be the early depletion of the most vulnerable cases who experience the event at an early phase as an unwanted effect of the invasive strategy. Therefore, we performed a landmark analysis using 1 year as the cut point based on the point where the Kaplan-Meier curves crossed.^10^

The RMST was used to analyze the primary end point and all the secondary end points. The treatment strategy indicator was modeled with time-dependent effects by including its interaction with restricted cubic splines on time. In addition, a robust variance estimation was performed for the within-cluster correlation of patients within centers. The analyses were adjusted for the cluster effect of the participating centers to account for potential variations in enrollment strategies, treatment practices, and other site-specific factors that could influence the outcomes. Using the same method, we also conducted a subgroup analysis based on the CFS as a measure of frailty (CFS score, 4 vs >4). Likewise, we performed a sensitivity analysis using inverse probability weighting on the propensity score to match patients within the CFS categories (CFS score, 4 vs >4). This propensity score included a robust set of baseline characteristics, ensuring a balanced comparative analysis. We applied a Royston-Parmar model with time-dependent effects and restricted cubic splines with 2 *df* to model the time-varying effect of the intervention accurately.

Analysis of rates of recurrent events was performed using bivcnto, a regression method suitable for analyzing correlated count outcomes.^11^ The aim was to test the effect of treatment on the rate of each recurrent event while adjusting the estimates for informative censoring due to death as a terminal event. Estimates are expressed as incidence rate ratios (IRRs) and 95% CIs. We also included robust variance estimation to account for within-cluster correlation.

A 2-sided *P* < .05 was considered statistically significant. All analyses were performed using Stata, version 17.0 (StataCorp LLC).

## Results

### Baseline Characteristics

The study population consisted of 167 patients (79 [47.3%] men and 88 [52.7%] women), with 84 allocated to the invasive group and 83 to the conservative group. All patients were White. Baseline characteristics were previously reported and can be found in eTable 1 in Supplement 2.^7^ The mean (SD) age was 86 (5) years. Baseline characteristics were well balanced between groups, except for a higher proportion of men (47 [56.6%] vs 32 [38.1%]; standardized mean difference, 0.40), previous myocardial infarction (32 [38.6%] vs 19 [22.6%]; standardized mean difference, 0.35), and previous percutaneous coronary revascularization (33 [39.8%] vs 19 [22.6%]; standardized mean difference, 0.37) in the conservative group compared with the invasive group. In the invasive group, 82 patients (97.6%) underwent a coronary angiogram. Conversely, 9 patients (10.8%) in the conservative group crossed over to invasive treatment because of recurrent ischemia (as prespecified in the study protocol). As a result, the initial revascularization rates were 50 (59.5%; complete revascularization in 27 [32.1%]) in the invasive group and 8 (9.6%; complete revascularization in 4 [4.8%]) in the conservative group.

The median follow-up in the total population was 1113 (IQR, 443-1441) days, and the median follow-up for surviving patients was 1424 (IQR, 1173-1592) days. No patients were lost to follow-up.

### Survival Outcomes

**Figure 1.**
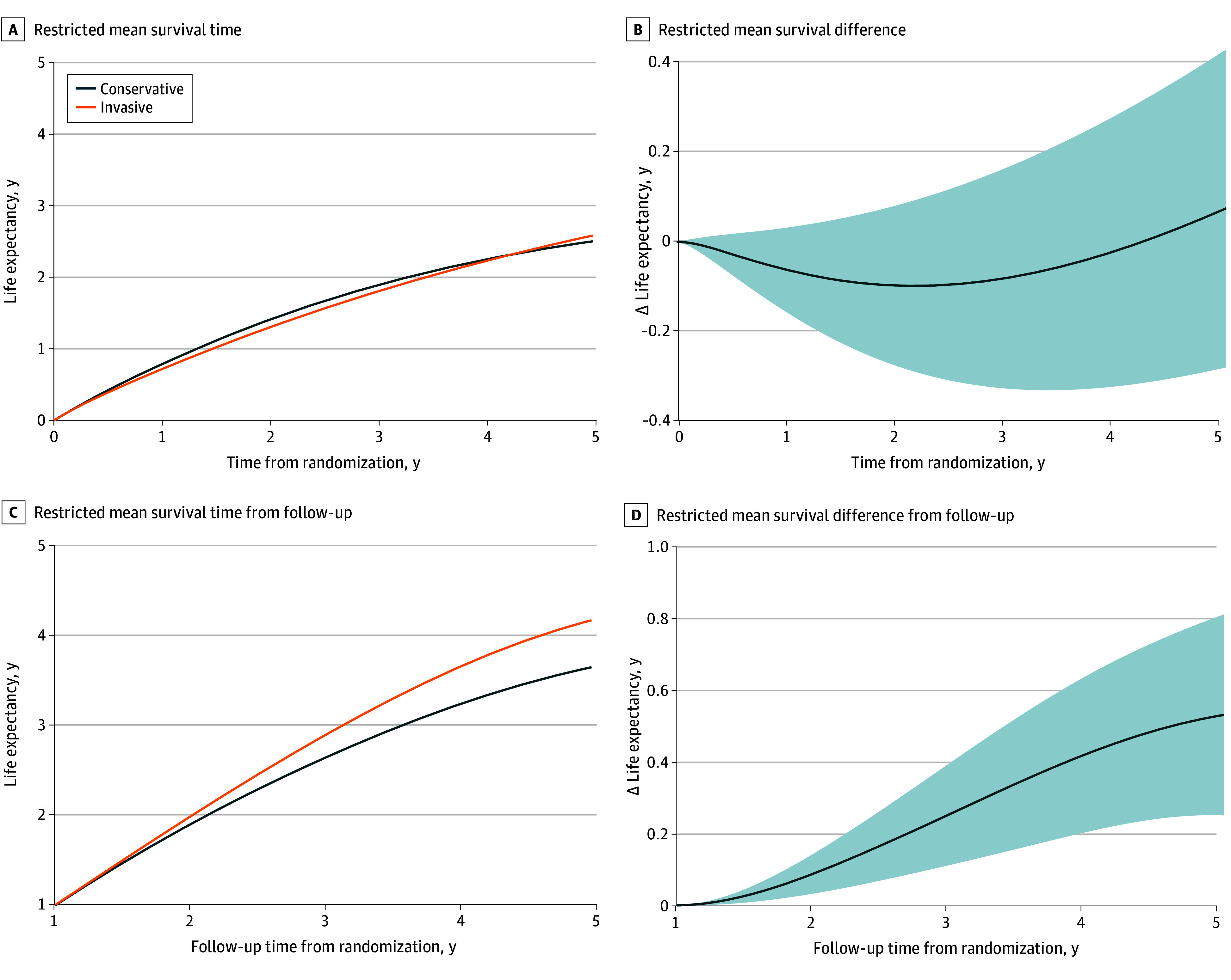
Restricted Mean Survival Curves for All-Cause Mortality Between Conservative and Invasive Treatment Strategies A and B, The follow-up period was initiated at randomization. C and D, The follow-up period was initiated 1 year after randomization. Shaded areas indicate the 95% CI.

A total of 93 patients died; 2 deaths in the invasive treatment group were related to percutaneous coronary intervention at the index hospitalization (one due to a complicated procedure and the other to renal failure after the procedure). The RMST for all-cause death over the entire follow-up was 3.13 (95% CI, 2.72-3.60) years in the invasive group and 3.06 (95% CI, 2.84-3.32) years in the conservative group. The RMST analysis showed inconclusive differences in survival time (invasive minus conservative group, 28 [95% CI, −188 to 230] days) (Figure 1A and B). However, patients who received invasive treatment tended to have shorter survival in the first year (invasive minus conservative, −28 [95% CI, −63 to 7] days), an effect that gradually neutralized later on. Indeed, invasive treatment significantly improved survival time in the landmark analysis after the first year (invasive minus conservative, 192 [95% CI, 90-230] days) (Figure 1C and D).

**Figure 2.**
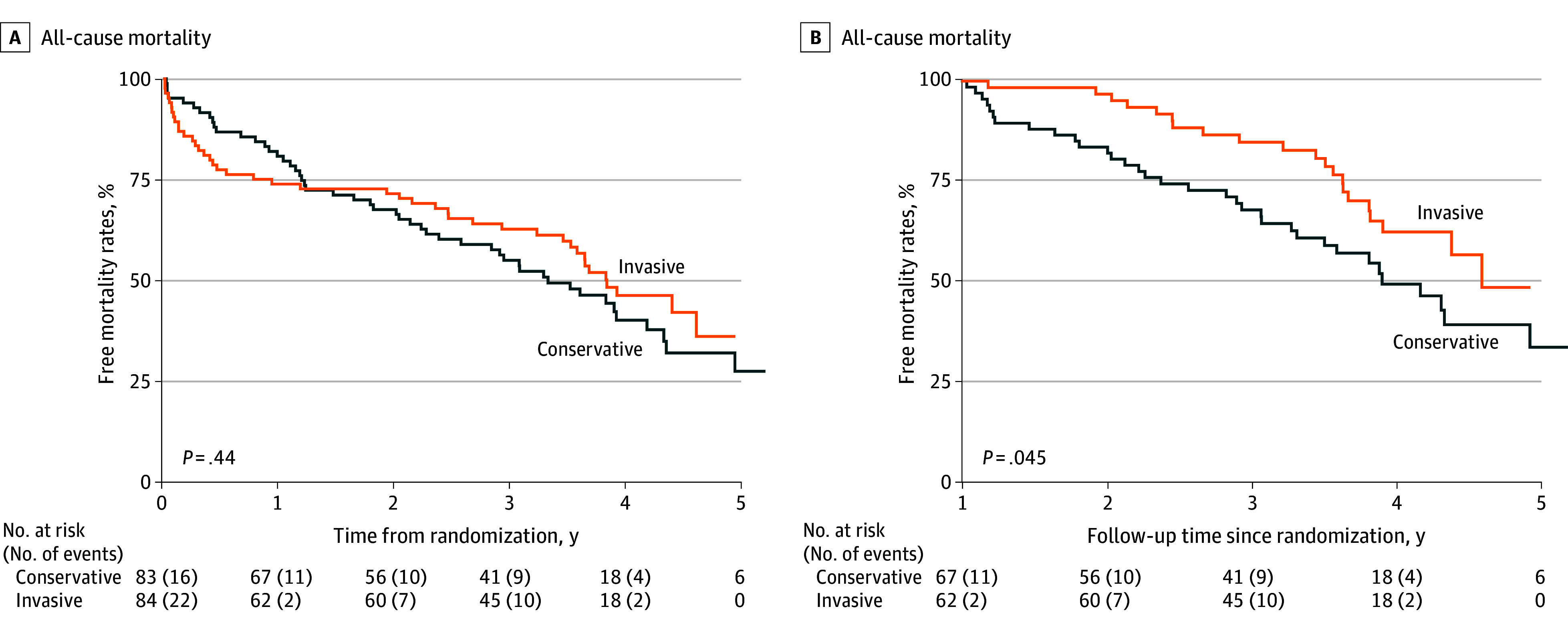
Kaplan-Meier Curves Comparing All-Cause Mortality Between Conservative and Invasive Treatment Strategies A, The follow-up period for the analysis was initiated at randomization. B, Thirty-eight patients were excluded due to early events. The follow-up period for the analysis was initiated 1 year after randomization.

The Kaplan-Meier curves showed no differences between the invasive and conservative strategies on mortality (hazard ratio, 0.85 [95% CI, 0.56-1.28]; *P* = .44, log-rank test) (Figure 2A). Notably, the curves crossed around 1 year, indicating a violation of the proportionality assumption for the treatment strategy. Specifically, the invasive approach appeared harmful within the first year, changing to a beneficial effect after the first year. In the landmark analysis starting from the first year of follow-up, the invasive treatment improved survival (hazard ratio, 0.58 [95% CI, 0.33-0.99]; *P* = .045, log-rank test) (Figure 2B).

Forty-nine deaths (52.7%) were noncardiac, 27 (29.0%) were cardiac, and 17 (18.3%) were of unknown causes (Table). eTable 2 in Supplement 2 provides information on the causes of noncardiac death. The leading cause related to invasive treatment was bleeding (5 deaths, 4 during the first year) compared with no deaths in the conservative treatment group.

**Table.** Distribution of Events During Follow-Up

Event	Treatment group, No./total No. (%) of events		
	Invasive	Conservative	All
Mortality	43/84 (51.2)	50/83 (60.2)	93/167 (55.7)
Noncardiac	27/84 (32.1)	22/83 (26.5)	49/167 (52.7)
Cardiac	12/84 (14.3)	15/83 (18.1)	27/167 (29.0)
Unknown	4/84 (4.8)	13/83 (15.7)	17/167 (18.3)
Readmission episodes^a^			
Cardiac causes			
All	81/179 (45.3)	73/188 (38.8)	154/367 (42.0)
Reinfarction	23/179 (12.8)	22/188 (11.7)	45/367 (12.3)
Revascularization	8/179 (4.5)	8/188 (4.3)	16/367 (4.4)
Unstable angina	4/179 (2.2)	8/188 (4.3)	12/367 (3.3)
Heart failure	39/179 (21.8)	30/188 (16.0)	69/367 (18.8)
Other cardiac reasons	7/179 (3.9)	5/188 (2.7)	12/367 (3.3)
Noncardiac causes			
All	98/179 (54.7)	115/188 (61.2)	213/367 (58.0)
Stroke	7/179 (3.9)	6/188 (3.2)	13/367 (3.5)
Bleeding	16 (8.9)	5/188 (2.7)	21/367 (5.7)
Other noncardiac reasons	75/179 (41.9)	104/188 (55.3)	179/367 (48.8)

^a^ Recurrent events included (n = 367).

### Subgroup Analysis

**Figure 3.**
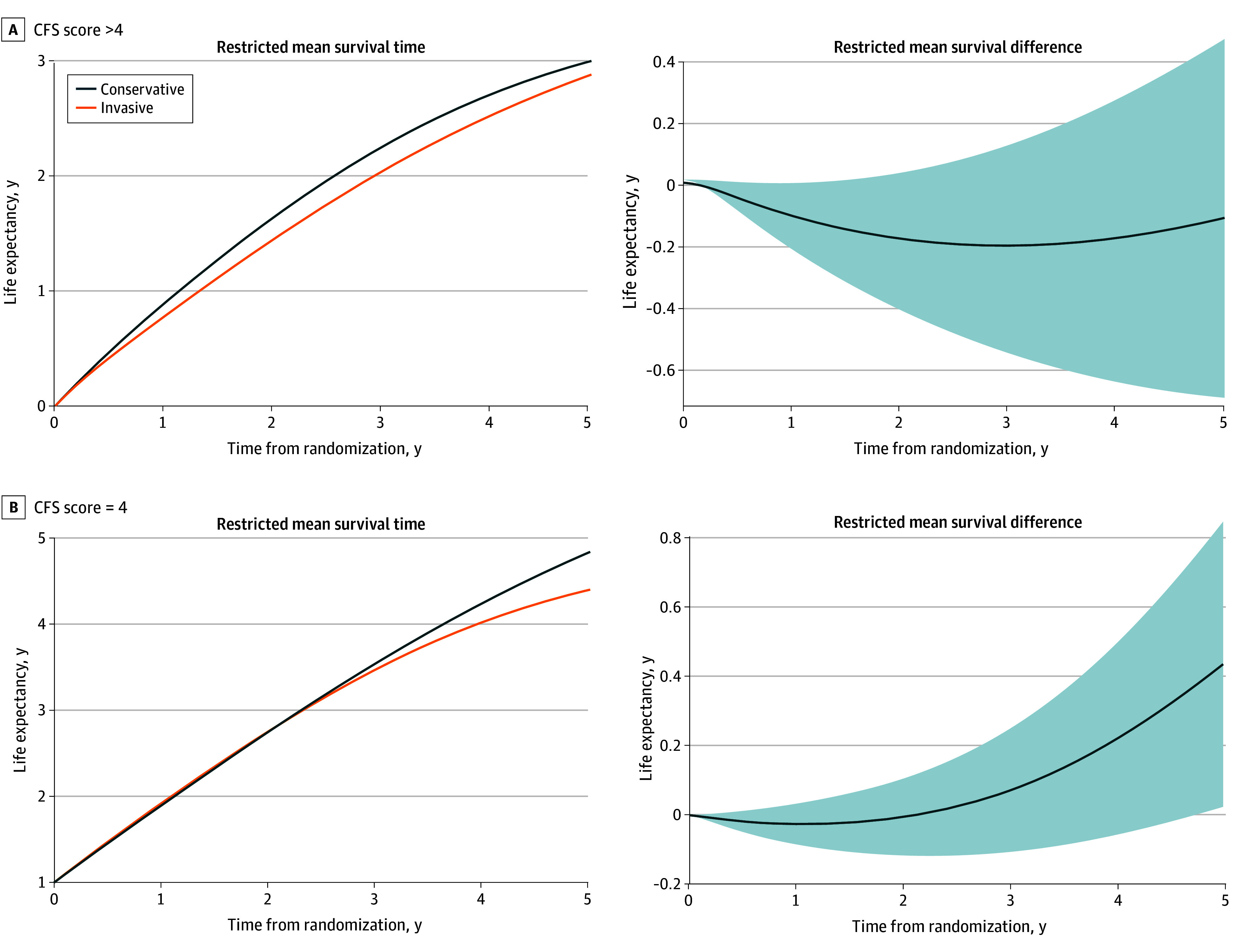
Restricted Mean Survival Curves for All-Cause Mortality Between Conservative and Invasive Treatment Strategies by Clinical Frailty Subgroups The subgroup with frailty was defined by a Clinical Frailty Scales (CFS) score of greater than 4; the vulnerable subgroup, a CFS score of 4. Shaded areas indicate the 95% CI.

The patient population was categorized into 2 subgroups based on their vulnerability^8^: the vulnerable subgroup, with a CFS of 4 (n = 43 [25.7%]), and the subgroup with frailty, with a CFS greater than 4 (n = 124 [74.3%]). The impact of invasive treatment differed between these 2 subgroups, as displayed in Figure 3. Within the subgroup with frailty (Figure 3A), invasive treatment significantly decreased survival during the first year. This effect changed over time, leading to nonsignificant differences at the end of the follow-up period (−43 [95% CI, −241 to 156] days). In contrast, within the vulnerable subgroup, invasive treatment was not associated with early hazard, resulting in more prolonged survival over the complete follow-up (difference, 160 [95% CI, 9-311] days). The results showed a significant effect of randomization (the intervention variable) on all-cause mortality in favor of the invasive strategy but only in a subset of vulnerable patients, with no effect on the subset of patients with frailty. Similar results were obtained using the propensity score model, ensuring a balanced comparative analysis (eFigure 2 in Supplement 2).

### Other Clinical Events

**Figure 4.**
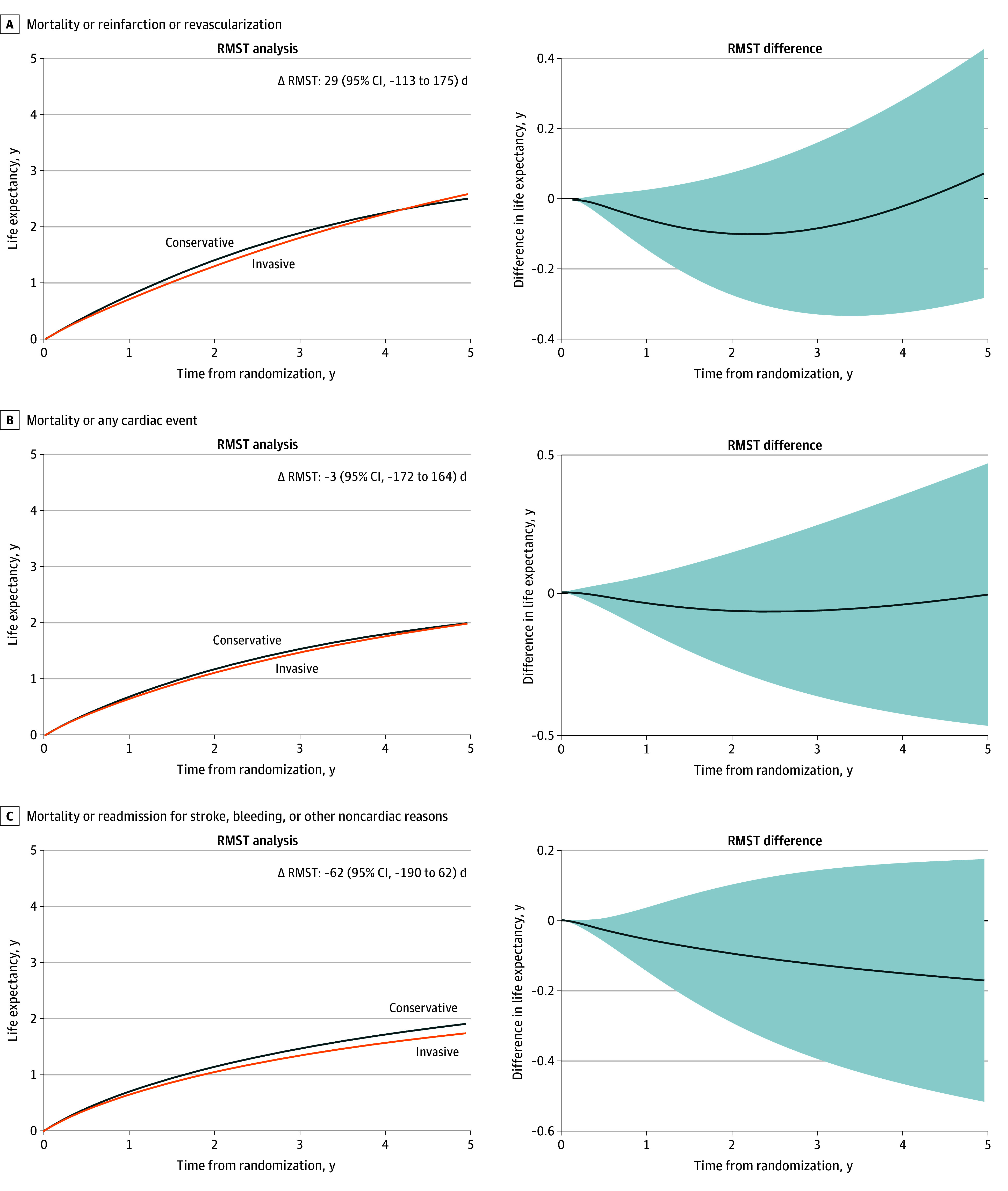
Restricted Mean Survival Curves Between Conservative and Invasive Treatment Strategies for the Secondary End Points The follow-up period was initiated at randomization. Cardiac events include reinfarction, revascularization, unstable angina, heart failure, or other cardiac reasons. RMST indicates restricted mean survival time. Differences in RMSTs are calculated as invasive minus conservative treatment. Shaded areas indicate the 95% CI.

There were 367 readmission episodes during the follow-up, including first-time and recurrent events (Table). Readmissions for noncardiac causes were more common. Figure 4 shows the differences in RMST for all secondary end points. There were no differences between invasive and conservative treatment for the composite secondary end points. Similarly, there were no significant differences for the individual components of the secondary end points considering recurrent events (eTable 3 in Supplement 2). The invasive treatment was associated with a numerically lower but nonsignificant risk of readmission for unstable angina and other cardiac reasons and a higher risk for bleeding.

## Discussion

This extended follow-up of a randomized clinical trial compared the midterm outcomes of invasive and conservative strategies in patients with frailty and NSTEMI. The main finding shows no differences in the number of days alive at the end of a median 1113-day follow-up. However, a distinctive course was observed with a change in direction, pointing to a reduced survival during the first year for patients who underwent invasive treatment, followed by a shift toward the opposite effect later.

The choice between invasive and conservative management strategies in patients with frailty and NSTEMI represents a clinical dilemma.^1^ Randomized clinical trials in older adults^3,4^ suggest that the benefit of invasive treatment is similar to that observed in younger individuals. However, patients with frailty or comorbidities are underrepresented in clinical trials. The burden of comorbidities may offset the potential benefit of an invasive strategy.^5,6^ The MOSCA-FRAIL randomized clinical trial explicitly focused on patients with frailty and found no differences between the invasive and conservative approaches at 1-year follow-up.^7^ In the present analysis, we confirm the lack of differences irrespective of treatment approach in the number of days alive or readmissions, for cardiac or noncardiac causes, in a median follow-up of 1113 days.

A notable finding is that invasive treatment reduced survival during the first year, particularly in patients with the most severe frailty. However, this outcome progressively decreased beyond the first year and shifted to a late benefit. Given their reduced life expectancy, the initial harm matters in patients with frailty. This survival time course may be attributed to a phenomenon in which a population exposed to an intervention is depleted of the most vulnerable cases who experience the event at an early phase.^12^ Once the susceptible individuals are removed from the population, the risk of the intervention decreases. The critical point is identifying susceptible patients to avoid early mortality risk. Patients with the highest levels of frailty (CFS >4) seem to be most susceptible in this case. The actual reasons for their susceptibility remain unknown, although we observed a higher rate of bleeding-related deaths and readmissions associated with the invasive strategy during the first year. On the other hand, the invasive strategy seemed to improve survival in patients with lower levels of frailty (CFS = 4); however, caution in interpreting this finding is warranted given the small number of patients in this subgroup.

The traditional primary end point of major adverse cardiac events used in clinical trials investigating invasive treatment may not be appropriate for patients with frailty.^13^ Noncardiac events exceeded cardiac events during the follow-up in this population, and this is a critical remark that should be considered in future studies, even for cardiac interventions. The MOSCA-FRAIL trial was designed for a primary end point of the number of days alive and out of the hospital during the first year. This end point is an alternative metric that encompasses both mortality and all hospitalizations and may best reflect the success of the treatment strategy.^14^ With the follow-up duration extending to a median of 1113 days, tracking hospitalization days became increasingly complex. Because the hazard proportionality assumption was not met in the extended follow-up analysis, we conducted the RMST analysis, which measures the mean event-free survival time up to a prespecified clinical point. The RMST difference represents the gain or loss in event-free survival time due to treatment compared with control.^9^ This difference may be more intuitive for the clinical communities.^15^

Defining frailty during hospitalization for acute NSTEMI is challenging, since most of the frailty scores were only validated in outpatient settings.^16^ Additionally, measured performance, such as gait speed and grip strength, can be impaired in acute illness and may not be evaluated or accurately reflect the baseline frailty status.^17^ Prior investigations have substantiated that frailty scales using questionnaires, such as the CFS, have proven to estimate mortality accurately among older patients who are hospitalized for acute illnesses.^17,18^

### Limitations

Several limitations merit acknowledgment. First, we recognize that the extended follow-up of the MOSCA-FRAIL trial adopted a study design wherein events were not centrally adjudicated. This approach raises the possibility of potential overestimation or underreporting of events. Local investigators were thoroughly trained and instructed to report and classify events to mitigate this risk. It is noteworthy that prior studies have demonstrated a high level of concordance between end points reported by investigators and those adjudicated centrally.^19,20^ Likewise, no information on the use of medications during follow-up was available. Second, the information about the total number of patients screened for enrollment was not collected. Enrollment was relatively slow, and not all consecutive patients were considered for randomization. On the other hand, the CFS score could be biased by subjective considerations. These facts could have led to a patient selection bias. Third, the wide 95% CI of RMST estimates underscores the inconclusiveness of these results, thus necessitating cautious interpretation. Therefore, our findings should be viewed as exploratory and hypothesis generating rather than conclusive. Fourth, the statistical power for subgroup analysis is limited, particularly in the vulnerable subgroup, hence these results should be interpreted with caution.

## Conclusions

In this extended follow-up of a randomized clinical trial of patients with frailty and NSTEMI who were clinically stable on admission, an initial invasive strategy did not yield conclusive midterm improvements compared with a conservative approach with watchful observation. However, there was a time-dependent pattern in the distribution of deaths between the treatment strategies. Specifically, the initial invasive treatment was associated with early harm during the first year, followed by a late benefit. This pattern suggests a phenomenon in which a susceptible population is depleted of the most vulnerable cases who experience an event at an early phase, which is particularly evident in patients with higher levels of frailty (CFS score >4). Therefore, an initial conservative strategy may be more appropriate for patients with NSTEMI and high levels of frailty. These findings provide valuable insights for clinical decision-making in this vulnerable patient population.

## Notes

Supplement 1.
Trial Protocol

Supplement 2. **eTable 1.** Baseline Patient Characteristics **eTable 2.** Causes of Noncardiac Death **eTable 3.** Effect of the Invasive Treatment on the Rate of Each Recurrent Event While Adjusting the Estimates for Informative Censoring Due to Death as a Terminal Event **eFigure 1.** CONSORT Flow Diagram **eFigure 2.** RMST Curve for All-Cause Mortality

Supplement 3.
Data Sharing Statement
