# Supplementary material for: Invasive Treatment Strategy in Adults With Frailty and Non–ST-Segment Elevation Myocardial Infarction: A Secondary Analysis of a Randomized Clinical Trial
Source: JAMA Netw Open. 2024 Mar 6;7(3):e240809. doi: 10.1001/jamanetworkopen.2024.0809 (PMC10918507; doi:10.1001/jamanetworkopen.2024.0809)

CONVOCATORIA PROYECTOS DE INVESTIGACIÓN EN SALUD
MEMORIA DE SOLICITUD

Expediente Nº

DATOS DEL INVESTIGADOR/A PRINCIPAL

APELLIDOS MIÑANA ESCRIVÁ **NOMBRE** GEMA
DNI/NIE: 20007045N **FECHA NACIMIENTO (DD/MM/AAAA):** 09/09/1978 **SEXO (V/M):** M
TELEFONO/S: 617551562 **ORCID:** 0000-0001-8777-2588

FORMACION ACADEMICA DEL INVESTIGADOR PRINCIPAL

LICENCIATURA/INGENIERIA/GRADO	CENTRO	FECHA
MEDICINA Y CIRUGIA	UNIVERSITAT DE VALENCIA	15/06/2002

DOCTORADO	CENTRO	FECHA
MEDICINA	UNIVERSITAT DE VALENCIA	03/04/2012

SITUACIÓN PROFESIONAL DEL INVESTIGADOR PRINCIPAL

POSICION ACTUAL F.E. CARDIOLOGÍA
FECHA INCORPORACION JUNIO 2015

ORGANISMO AGENCIA VALENCIANA DE SALUT

CENTRO/FACULTAD/ESCUELA/INSTITUTO:
HOSPITAL CLÍNICO UNIVERSITARIO DE VALENCIA. UNIVERSITAT DE VALENCIA

DEPT./UNIDAD/SECC.: CARDIOLOGÍA

CORREO/S ELECTRONICO/S: gemineta@gmail.com

VINCULACIÓN LABORAL
- ○ FUNCIONARIO/A
- ○ ESTATUTARIO/A
- ◉ LABORAL FIJO O INDEFINIDO
- ○ LABORAL TEMPORAL
- ○ EMÉRITO

ACTIVIDADES ANTERIORES DE CARÁCTER CIENTÍFICO O PROFESIONAL

AÑOS INICIO-FINAL	PUESTO	INSTITUCIÓN
2003-2008	MIR CARDIOLOGÍA	HOSPITAL CLÍNICO UNIV. VALENCIA
2008-2011	F.E. CARDIOLOGÍA	HOSPITAL CLÍNICO UNIV. VALENCIA
2011-2015	F.E. CARDIOLOGÍA	HOSPITAL DE MANISES
2015-	F.E. CARDIOLOGÍA	HOSPITAL CLÍNICO UNIV. VALENCIA
2005-2011	COLABORADOR DOCENTE	UNIVESITAT DE VALENCIA
2011-2012	PROFESOR ASOCIADO ASISTENCIAL	UNIVESITAT DE VALENCIA
2012-2015	PROFESOR ASOCIADO DE PRÁCTICAS	UNIVERSIDAD CATOLICA VALENCIA
2015-	PROFESOR ASOCIADO ASISTENCIAL	UNIVESITAT DE VALENCIA

Expediente Nº

TITULO: **Randomized comparison between the invasive and conservative strategies in elderly frail patients with non-ST elevation myocardial infarction: The MOSCA-FRAIL Clinical Trial**

INVESTIGADOR/A PRINCIPAL: Gema Miñana Escrivá

CO-INVESTIGADOR/A PRINCIPAL:

TIPO DE PROYECTO: ○ **INDIVIDUAL** ○ **COORDINADO** ◉ **MULTICÉNTRICO**

NOMBRE DEL IP COORDINADOR:
(Cumplimentar sólo en caso de proyectos coordinados)

DURACION: ☒ **3 AÑOS**

RESUMEN (Objetivos y Metodología del Proyecto) **(Ajústese al espacio disponible)**

Current guidelines recommend an invasive strategy in most patients with non ST segment elevation myocardial infarction (NSTEMI). Frail elderly patients are commonly excluded from clinical trials. Therefore, the applicability of these recommendations in patients at older ages remains controversial. Elderly patients are commonly managed conservatively in routine clinical practice. The hypothesis of this project is that an invasive strategy may improve prognosis in frail elderly patients with NSTEMI. This is a multicenter randomised trial designed to compare an invasive strategy with a conservative approach in elderly patients with NSTEMI and frailty criteria. Inclusion criteria will be: diagnosis of NSTEMI, age 70 years or older and frailty criteria as defined by the Clinical Frailty Scale (≥ 4 points). Patients will be randomized within the first 48 hours of admission to an invasive strategy (coronary angiography and revascularisation if indicated according to coronary anatomy) or a conservative approach (medical treatment and angiography only in cases of refractary ischemia or clinical unstabilisation). Main outcome measure will be the number of days alive out of the hospital (NDAOH) during the first year after the admission. Coprimary endpoint will be time to cardiovascular death o reinfarction during the first year. Sample size will be 176 patients (88 in each arm), assuming an increase in 20% in the NDAOH during the first year. Reporting a benefit by the invasive strategy in these patients might lead to important healthcare, s

TITLE: Randomized comparison between the invasive and conservative strategies in elderly frail patients with non-ST elevation myocardial infarction: The MOSCA-FRAIL Clinical Trial

ABSTRACT (Objectives and Methodology of the project) **(Please only use the space provided below)**

Current guidelines recommend an invasive strategy in most patients with non ST segment elevation myocardial infarction (NSTEMI). Frail elderly patients are commonly excluded from clinical trials. Therefore, the applicability of these recommendations in patients at older ages remains controversial. Elderly patients are commonly managed conservatively in routine clinical practice. The hypothesis of this project is that an invasive strategy may improve prognosis in frail elderly patients with NSTEMI. This is a multicenter randomised trial designed to compare an invasive strategy with a conservative approach in elderly patients with NSTEMI and frailty criteria. Inclusion criteria will be: diagnosis of NSTEMI, age 70 years or older and frailty criteria as defined by the Clinical Frailty Scale (≥ 4 points). Patients will be randomized within the first 48 hours of admission to an invasive strategy (coronary angiography and revascularisation if indicated according to coronary anatomy) or a conservative approach (medical treatment and angiography only in cases of refractary ischemia or clinical unstabilisation). Main outcome measure will be the number of days alive out of the hospital (NDAOH) during the first year after the admission. Coprimary endpoint will be time to cardiovascular death o reinfarction during the first year. Sample size will be 176 patients (88 in each arm), assuming an increase in 20% in the NDAOH during the first year. Reporting a benefit by the invasive strategy in these patients might lead to important healthcare, social and economic implications.

Expediente Nº	

INVESTIGADOR/A PRINCIPAL: Gema Miñana Escrivá

MEMORIA DE SOLICITUD PROYECTO DE INVESTIGACIÓN EN SALUD
SECCIÓN ANTECEDENTES Y ESTADO ACTUAL DEL TEMA

Finalidad del proyecto, antecedentes y estado actual de los conocimientos científico-técnicos, grupos nacionales o internacionales que trabajan en la línea específica del proyecto o en líneas afines.
Citar las referencias en el apartado siguiente: Bibliografía más relevante. **Máximo 3 páginas (15.700 caracteres)**

Frailty can be defined as a physiological state of decreased resistance to stressors that results from diminished physiological reserves of multiple systems and causes increased vulnerability to adverse outcomes (1). Acute coronary syndromes (ACS) can be major stressful events for older patients. The prevalence of frailty in patients admitted for ACS ranges from 27% to 34% (2-4). The presence of frailty and pre-frailty identifies patients at increased risk of mortality after hospitalization for ACS. This may be due in part to the underutilization of therapies recommended by clinical practice guidelines in frail patients. These patients are also less likely to be admitted to cardiac intensive care units (coronary units) (5-7), have a longer hospital stay (5, 6, 8), and are more likely to have readmissions for cardiovascular events (6, 7, 9).

In series of patients with non-ST-segment elevation myocardial infarction (NSTEMI), frailty predicts the risk of 30-day and mid-term mortality and cardiovascular events independently of comorbidities and other risk factors (5, 6, 10). In another study, which analyzed a wide range of geriatric conditions (physical disability, instrumental disability, cognitive impairment, and comorbidities), frailty captured most of the prognostic information derived from these conditions (3, 4).Translated with www.DeepL.com/Translator (free version)

There is currently a lack of evidence regarding the best management of frail patients with NSTEMI. Some studies have shown lower rates of prescribing recommended drugs (angiotensin-converting enzyme inhibitors (ACE inhibitors), angiotensin II receptor antagonists (ARBs), and statins) after ACS (10, 11)). Prescribing decisions are especially complex for frail patients because of their increased risk of side effects. Therefore, careful clinical judgment is required to weigh the balance between risk and benefit, taking into account the burden of polypharmacy and overall life expectancy. Along the same lines, the bleeding risk associated with dual antiplatelet therapy is associated with age (12), its association with frailty being more controversial (10). Overall, less potent antiplatelet agents tend to be used in elderly or frail patients. Similarly, drug-eluting stents have been used less frequently to date in this subgroup because of the need for longer duration of dual platelet antiplatelet aggregation. However, new designs of drug-eluting stents allow a short period of dual platelet antiplatelet aggregation and may be a very useful tool in these patients (13).

Clinical practice guidelines recommend a routine invasive strategy in NSTEMI in most patients (14), although elderly patients and those with comorbidities are often severely underrepresented in clinical trials supporting such recommendations. Similarly, patients with frailty criteria are often excluded from randomized trials (10). In routine clinical practice, invasive management is clearly less used in frail patients and in those with comorbidities (5, 8, 15)). Moreover, frail patients undergoing coronary angiography have lower revascularization rates than nonfrail patients (5, 8). This may be, in part, a reflection of more complex coronary artery disease, which may not be amenable to percutaneous coronary intervention (PCI) (16). However, it is also possible that the lower rates of revascularization in these patients reflect a perception of increased risk from the intervention and a lower expectation of benefit. On the other hand, after percutaneous or surgical revascularization, frailty and comorbidity are associated with adverse long-term outcomes (16,17)). However, several registries have shown a benefit associated with in-hospital revascularization in comorbid patients with NSTEMI (18,19)).

Some clinical trials addressed the role of invasive strategy in elderly patients, showing mixed results. A routine invasive strategy was not statistically superior to a selective invasive strategy in elderly patients with NSTEMI (20), but that study was underpowered because of its small sample size. The randomized "After Eighty" trial included 457 patients older than 80 years with NSTEMI and demonstrated the benefit of the invasive strategy in reducing the composite event of death or cardiovascular events at 1.5 years (21). Of note, no patient underwent cardiac catheterization under any circumstances in the conservative arm of that study. In addition, only 23% of potential candidates for inclusion were ultimately randomized, suggesting a bias toward lower-risk patients. Recently, the

randomized MOSCA trial evaluated the efficacy of an invasive strategy in elderly patients with NSTEMI and associated comorbidities (22). Although this was a small trial, the invasive strategy reduced the likelihood of death or ischemic events at 3 months. However, this benefit faded at 2.5 years of follow-up. In none of the aforementioned studies was a formal assessment of frailty or other variables related to aging (disability, cognitive status, nutritional risk, quality of life) performed. To date, no randomized clinical trial has addressed the role of the invasive strategy in patients with NSTEMI and frailty criteria.

On the other hand, although most studies have focused primarily on events such as death, myocardial infarction, need for revascularization or readmission, other factors such as patients' need to regain independent living and return to their previous life should also be taken into account. The presence of geriatric syndromes (including frailty, cognitive impairment, severe dependency and depression) is not only associated with worse clinical outcomes, but with a higher risk of functional decline and the need for new social support, i.e., a higher level of dependency. This has a remarkable impact on the patient's quality of life and psychological well-being, but also frequently becomes a heavy social and economic burden for patients and their relatives. Therefore, one of the real challenges in the management of ACS in very elderly patients is the prevention of dependency. In this regard, the use of new target variables in clinical trials or registries, especially aimed at measuring the level of independence and quality of life, is particularly important (23).

The role of a routine invasive strategy in frail patients is currently uncertain. We hypothesize that a routine invasive strategy in frail patients with NSTEMI will improve outcomes. The aim of the trial is to evaluate the efficacy and safety of a routine invasive strategy to increase hospitalization-free survival during the first year and improve cardiovascular outcomes.

Expediente Nº	
**INVESTIGADOR/A PRINCIPAL:** Gema Miñana Escrivá	

MEMORIA DE SOLICITUD PROYECTO DE INVESTIGACIÓN EN SALUD
SECCIÓN ANTECEDENTES Y ESTADO ACTUAL DEL TEMA

Citar las referencias incluidas en el apartado anterior: Antecedentes y Estado actual. (Máximo 1 página)

1. Fried LP, et al. Untangling the concepts of disability, frailty, and comorbidity: implications for improved targeting and care. J Gerontol A Biol Sci Med Sci. 2004;59:255-63.
2. Purser JL, et al. Identifying frailty in hospitalized older adults with significant coronary artery disease. J Am GeriatrSoc 2006;54:1674-81
3. Sanchis J, et al. Frailty and other geriatric conditions for risk stratification of older patients with acute coronary syndrome. Am Heart J. 2014;168:784-91.
4.Sanchis J, et al. Prognostic value of geriatric conditions beyond age after acute coronary syndromes. Mayo Clinic Proceed 2017 (in press)
5. Ekerstad N, et al. Frailty is independently associated with short-term outcomes for elderly patients with non-ST-segment elevation myocardial infarction. Circulation 2011;124:2397-404
6. Ekerstad N, et al. Frailty is independently associated with 1-year mortality for elderly patients with non-ST-segment elevation myocardial infarction. Eur J PrevCardiol 2014;21:1216-24.
7. Myers V, et al. Clinical relevance of frailty trajectory post myocardial infarction. European Journal of Preventive Cardiology 2014; 21: 758-66.
8. Graham MM, et al. Frailty and outcome in elderly patients with acute coronary syndrome. Can J Cardiol. 2013;29:1610-5
9. Kang L, et al. Is frailty associated with short-term outcomes for elderly patients with acute coronary syndrome? Journal of Geriatric Cardiology. 2015; 12: 662-7.
10. White HD, et al; TRILOGY ACS investigators. Frailty is associated with worse outcomes in non-ST-segment elevation acute coronary syndromes: Insights from the TaRgeted platelet Inhibition to cLarify the OptimalstrateGy to medicallY manage Acute Coronary Syndromes (TRILOGY ACS) trial. Eur Heart J Acute Cardiovasc Care 2016;5:231-42.
11. Matsuzawa Y, et al. Association between gait speed as a measure of frailty and risk of cardiovascular events after myocardial infarction. J Am Coll Cardiol. 2013;61:1964-72.
12. Roe MT, et al. Elderly patients with acute coronary syndromes managed without revascularization: insights into the safety of long-term dual antiplatelet therapy with reduced-dose prasugrel versus standard-dose clopidogrel. Circulation 2013;128:823-33
13. Urban P, et al; LEADERS FREE Investigators. Polymer-free Drug-Coated Coronary Stents in Patients at High Bleeding Risk. N Engl J Med. 2015;373:2038-4711.
14. Roffi M, et al. 2015 ESC Guidelines for the management of acute coronary syndromes in patients presenting without persistent ST-segment elevation: . Eur Heart J 2016;37:267-315
15.Sanchis J, et al. Influence of comorbid conditions on one-year outcomes in non-ST-segment elevation acute coronary syndrome. Mayo Clin Proc 2011;86:291-6
16. Singh M, et al. Influence of frailty and health status on outcomes in patients with coronary disease undergoing percutaneous revascularization. CircCardiovascQual Outcomes. 2011;4:496-502
17. Sündermann S, et al. Comprehensive assessment of frailty for elderly high-risk patients undergoing cardiac surgery. Eur J CardiothoracSurg 2011;39:33-7.
18. Bauer T, et al; Acute Coronary Syndromes Registry (ACOS) Investigators. Effect of an invasive strategy on in-hospital outcome in elderly patients with non-ST-elevation myocardial infarction. Eur Heart J. 2007; 28:2873-8
19. Palau P, et al. Differential prognostic effect of revascularization according to a simple comorbidity index in high-risk non-ST-segment elevation acute coronary syndrome. ClinCardiol 2012;35:237-43
20. Savonitto S, et al; Italian Elderly ACS Trial. Early aggressive versus initially conservative treatment in elderly patients with non-ST-segment elevation acute coronary syndrome: a randomized controlled trial. JACC Cardiovasc Interv 2012;5:906-16
21. Tegn N, et al; After Eighty study investigators. Invasive versus conservative strategy in patients aged 80 years or older with non-ST-elevation myocardial infarction or unstable angina pectoris (After Eighty study): an open-label randomised controlled trial. Lancet. 2016;387:1057-65
22. Sanchis J, et al. Randomized comparison between the invasive and conservative strategies in comorbid elderly patients with non-ST elevation myocardial infarction. Eur J Intern Med. 2016;35:89-94.
23. Montilla Padilla I, et al. Management of Acute Coronary Syndromes in Geriatric Patients. Heart Lung Circ. 2017;26:107-113.

Expediente Nº

INVESTIGADOR/A PRINCIPAL: Gema Miñana Escrivá

MEMORIA DE SOLICITUD PROYECTO DE INVESTIGACIÓN EN SALUD
SECCIÓN HIPÓTESIS Y OBJETIVOS

(Ajústese al espacio disponible)

HIPÓTESIS

In patients older than 70 years with non-ST-segment elevation myocardial infarction (NSTEMI) and with frailty criteria, the application of an invasive strategy will improve prognosis compared to a conservative strategy. This improvement will translate into longer hospitalization-free survival in the first year after admission and a lower probability of cardiovascular events during the first year after NSTEMI.

OBJETIVOS

Primary objective:
1.1 To study the effect of invasive strategy on the possible increase in the number of live days out of Hospital during the first year in elderly patients with NSTEMI and frailty criteria.

Co-primary objective:

1.2 To study the effect of the invasive strategy on the incidence of cardiovascular death or myocardial infarction during the first year in elderly patients with NSTEMI and frailty criteria.

Secondary objectives:

To study, in elderly patients with NSTEMI and frailty criteria, the impact of the invasive strategy on the following variables.

2.1 All-cause death.

2.2 Cardiovascular death.

2.3 Myocardial infarction.

2.4 Stroke.

2.5 Readmissions due to cardiac and extracardiac causes.

2.6 Hemorrhagic events (only bleeding type ≥2 as defined by the Bleeding Academic Research Consortium will be considered).

Expediente Nº	

INVESTIGADOR/A PRINCIPAL: Gema Miñana Escrivá

MEMORIA DE SOLICITUD PROYECTO DE INVESTIGACIÓN EN SALUD
SECCIÓN PROYECTOS COORDINADOS

En caso de Proyectos Coordinados, el COORDINADOR deberá indicar:
- Objetivos globales del proyecto coordinado, la necesidad de dicha coordinación y el valor añadido que se espera obtener de la misma.
- Objetivos específicos de cada subproyecto (deben están recogidos además en la memoria de cada subproyecto)
- Interacción entre los distintos objetivos, actividades y subproyectos.
- Los mecanismos de coordinación previstos para la eficaz ejecución del proyecto. **Máximo 3 páginas (15.700 caracteres)**

NA

Expediente Nº	

INVESTIGADOR/A PRINCIPAL: Gema Miñana Escrivá

MEMORIA DE SOLICITUD PROYECTO DE INVESTIGACIÓN EN SALUD
SECCIÓN METODOLOGÍA
Diseño, sujetos de estudio, variables, recogida y análisis de datos y limitaciones del estudio.
Máximo 3 páginas (15.700 caracteres)

STUDY DESIGN

Prospective randomized multicenter study (1:1) to compare an invasive versus conservative strategy in patients with NSTEMI older than 70 years with frailty criteria.
The study will be carried out in 13 hospitals with the participation of clinical and interventional cardiologists from the participating centers. The study will be carried out following a single clinical protocol that must be approved by the Clinical Research Ethics Committees of the participating hospitals. In the development of the protocol, the principles enunciated in the Declaration of Helsinki (2013, Brazil), as well as the standards of Good Clinical Practice, have been taken into account. The development of the study will be coordinated by the two principal investigators of the study, Dr. Albert Ariza-Solé, PI of the Hospital Universitario de Bellvitge and Dr. Gema Miñana, PI of the Hospital Clínico Universitario de Valencia.

PATIENT POPULATION

Inclusion criteria:
Patients who meet the following three inclusion criteria will be eligible:
1) NSTEMI, defined by acute chest pain and troponin elevation (according to the local laboratory troponin assay), in the absence of ST-segment elevation on the electrocardiogram.
2) Age ≥ 70 years.
3) Frailty criteria defined by ≥4 points on the Clinical Frailty Scale (Rockwood K, et al. CMAJ. 2005;173:489-95).

Exclusion criteria:
1)Previously known non-revascularizable coronary artery disease.
2)Significant concomitant non-ischemic heart disease (severe valvular heart disease, hypertrophic cardiomyopathy...).
3)Inability to understand or sign the informed consent.
4) Life expectancy <12 months.

In addition to the defined inclusion and exclusion criteria, patient inclusion in the study should be considered reasonable in the judgment of the cardiologist responsible for the patient. Reasons for considering inappropriate participation could be consideration of the clinical need for invasive management or the presence of any factor that makes the invasive strategy not a reasonable alternative. All potentially eligible patients (i.e., those meeting all inclusion criteria and no exclusion criteria) ultimately not included in the trial due to a medical decision will be registered in parallel in a database and followed up.

PROTOCOL

Patients will be randomized within 48 hours of admission to undergo coronary angiography and coronary revascularization as deemed appropriate during the index hospitalization or be treated with medical therapy. Patients will be assigned to both treatment groups using a computer-generated randomization scheme to assign participants in a 1:1 ratio. Randomization will be through a website where the process will be hidden from the investigators until interventions are assigned. Medical treatment at admission and discharge will be optimized according to clinical practice guidelines in both treatment arms.

In the invasive management group the patient will undergo coronary angiography (and coronary revascularization as deemed appropriate) within 72 hours of admission. When performing percutaneous coronary interventionism

(PCI), the type of stent implanted will be at the discretion of the treating cardiologist, although drug-eluting stents that have shown safety using a period of short-term antiplatelet therapy will be recommended to reduce the potential risk of bleeding in this population. In contrast, patients assigned to the noninvasive group will receive only medical therapy, although cardiac catheterization will be allowed in case of poor in-hospital outcome due to recurrent ischemia or clinical instability during index admission.

The pre-discharge left ventricular ejection fraction (LVEF) will be determined in all patients by echocardiography (Philips iE-33). The standard duration of dual antiplatelet therapy will be 1 year in both study arms, although in patients at high bleeding risk (at the discretion of the treating physician) or in need of oral anticoagulation, one antiplatelet drug may be withdrawn after the first month. Detailed clinical and treatment data will be collected on admission and during hospitalization. In addition, a thorough geriatric assessment will be carried out prior to discharge by interviewing the patient and/or family or caregivers, with reference to the patient's condition prior to admission.

PROCEDURES

In-hospital geriatric assessment: This will be carried out by local investigators, duly advised by the Hospital de Bellvitge team (Dr Albert Ariza-Solé, Dr Francesc Formiga).

1. Functional capacity for basic activities of daily living will be assessed using the Barthel Index (Mahoney FI, et al. Md State Med J. 1965;14:61-65). This is an ordinal scale with a total score from 0 to 100, where intermediate ranges help to assess different degrees of dependency: total (0-20), severe (21-40), moderate (41-60), mild (61-90), and independent (>90). Instrumental activities will be assessed using the Lawton-Brody Index (Lawton MP, et al. Gerontologist. 1969;9:179-186).

2. Cognitive status will be assessed by the Pfeiffer Test (Pfeiffer E. J Am Geriatr Soc. 1975;23:433 -441).

3. Previous frailty will be assessed, in addition to the Rockwood scale, by the FRAIL scale (Abellan van Kan G, et al. J Nutr Health Aging. 2008;12:29-37), which includes assessment of fatigue, endurance, ambulation, concomitant diseases and weight loss. After the acute phase (once the patient is stabilized), the Short Physical Performance Battery (SPPB) will be performed (Guralnik JM, et al. J Gerontol A Biol Sci Med Sci. 2000;55:M221-31). This test includes: a) balance in three positions (equal feet, semi-tandem, and tandem), b) walking speed (4 meters lengthwise), and c) standing and sitting in a chair five times. The total SPPB score is the sum of the three sub-tests, ranging from 0 (worst) and 12. A score below 10 indicates weakness and an increased risk of disability and falls.

4. For the evaluation of comorbidity, the Charlson index will be applied, with a maximum score of 37 points (Charlson ME, et al. J Chronic Dis. 1987; 40:373-383). The number of chronic prescription drugs taken by the patient prior to admission will also be collected.

5. Nutritional risk assessment will be performed using the Mini Nutritional Assessment-Short Form (MNA-SF) (Rubenstein LZ, et al. J Gerontol A Biol Sci Med Sci. 2001;56:M366-72), whose value ranges from 0 to 14 points. Scores below 11 identify patients at risk for malnutrition.

6. Quality of life will be analyzed using the European Quality of Life Five Dimension Five Level Scale (EQ-5D-5L) test (Buanes EA, et al. Resuscitation. 2015;89:13-8).

ENDPOINTS

1.1 The primary endpoint will be the number of days alive free of hospitalization during the first year.
1.2 The co-primary endpoint will be the time to the first occurrence of one of the events included in the combined event (cardiovascular death or myocardial infarction).
Secondary endpoints include time to presentation of:
2.1 All-cause death.
2.2 Cardiovascular death.
2.3 Myocardial infarction.
2.4 Stroke.
2.5 Readmission due to cardiac and extracardiac causes,
2.6 Hemorrhagic events and cerebrovascular accidents.

Only bleeding type ≥2 as defined by the Bleeding Academic Research Consortium (BARC) will be considered (Mehran R, et al. Circulation. 2011;123:2736-47). The incidence of procedure-related myocardial infarction will be assessed by measuring troponins 12 hours after revascularization. Procedure-related infarction will be defined as troponin elevation >5x the 99th percentile after percutaneous coronary intervention and >10x the 99th percentile after coronary surgery, plus data suggestive of acute ischemia based on clinical symptoms, electrocardiogram, angiography, or imaging techniques.
Geriatric evaluation during follow-up (to be performed by local investigators, duly advised by the Hospital de Bellvitge team (Dr Albert Ariza-Solé, Dr Francesc Formiga)): Functional capacity (Barton Index), instrumental activities (Lawton-Brody Index), cognitive capacity (Pfeiffer Test) and quality of life (EQ-5D-5L) will be reassessed at 6 months.
Clinical follow-up will include a clinic visit or telephone contact at 3 months and 1 year.

STUDY COMMISSIONS

The present project is an independent, non-industry funded clinical trial, driven as an initiative of the investigator team. A steering committee will be responsible for overseeing the scientific and operational aspect of the study. Patients and investigators will not be masked to treatment allocation, but clinical events will be evaluated by an event allocation committee blinded to the treatment arm to prevent bias. Similarly, a data safety monitoring board, which will not be aware of patient treatment assignment, will be responsible for making recommendations to the steering committee regarding endpoints and any potential significant observations related to patient safety.

STATISTICAL ANALYSIS AND SAMPLE SIZE CALCULATION

- Sample Size Calculation

There are no data on the best management of frail patients with NSTEMI. In a previous study involving elderly patients with NSTEMI and comorbidities, those patients undergoing a conservative strategy had a mean of 273 days out of hospital (standard deviation of 123 days) during the first year after discharge (Sanchis J, et al. Eur J Intern Med. 2016;35:89-94). Assuming that frail patients could have a similar profile, and considering a 20% increase in the proportion of out-of-hospital alive days (55 days) with an invasive strategy, with a statistical power of 80%, a bilateral alpha error of 0.05 and 10% loss to follow-up, we estimated a sample size of 176 patients (88 per arm).

A multicenter approach will be required to achieve the estimated sample size.

The following hospitals have committed to participate: Clínico Valencia (Gema Miñana, Juan Sanchis, Paolo Racugno and Martina Amiguet), Bellvitge Barcelona (Albert Ariza-Solé and JA Gómez Hospital), Moisès Broggi St Joan Despí (Antoni Carol), Vall d'Hebron Barcelona (JA Barrabes and Bruno García del Blanco), Germans Trias i Pujol Badalona (Cinta Llibre, Eduard Fernandez), Sant Pau Barcelona (Alex Sionis, Juan García Picart), Gregorio Marañon Madrid (Manuel Martínez Sellés, Jaime Elízaga), Arganda (Adolfo Villa), 12 de Octubre Madrid (Roberto Martín, Felipe Hernández) La Princesa Madrid (Pablo Díez Villanueva, Fernando Alfonso), Ramon y Cajal Madrid (Marcelo Sanmartin, Rosana Hernández-Antolín) Cunqueiro Vigo (Emad Abu Assi, Jose Antonio Baz), Virgen Arrixaca Murcia (Francisco Marin, Eduardo Pinar).

- Statistical analysis

All statistical comparisons will be made on an intention-to-treat basis. Results will be presented as frequencies or mean (standard deviation), as appropriate. Comparisons between groups will be made using the t-test or Fisher's exact test.

Patient follow-up will be censored at the time of death or at the end of the study. The primary endpoint, as a continuous variable, will be compared between the two groups using the ANOVA test. Regarding the co-primary endpoint and secondary endpoints, the effect of the invasive strategy on clinical events will be described by a Kaplan-Meier method and evaluated using a Cox regression model. Hazard ratio (HR) and 95% confidence intervals (CI) will be calculated. A pre-established subgroup analysis will be performed according to comorbidities (Charlson

index).

For all analyses, a 2-tailed p-value of <0.05 will be considered statistically significant.

Expediente Nº

INVESTIGADOR/A PRINCIPAL: Gema Miñana Escrivá

MEMORIA DE SOLICITUD PROYECTO DE INVESTIGACIÓN EN SALUD
SECCIÓN PLAN DE TRABAJO

Etapas de desarrollo y distribución de las tareas de todo el equipo investigador, y las asignaciones previstas para el personal técnico que se solicita. Indicar además el lugar/centro de realización del proyecto.

(Ajústese al espacio disponible)

Initiation and start-up phase (1-2 months)
During the initiation and start-up phase, the protocol will be drawn up in accordance with the applicable regulations and clinical practice guidelines, incorporating the specific data collection notebook for the study, which will include detailed information on the study variables, the patient information sheet and informed consent. This activity will be carried out by the 2 centers leading the project through the PIs responsible, Dr Albert Ariza-Solé and Dr Gema Miñana, with the support of the advisory team composed of Drs J. Sanchis and H. Bueno.
During the development of the study, the research team will have the management support of UICEC-IDIBELL and INCLIVA, which are part of the Platform of Clinical Trial Units of the Research Institutes of the Bellvitge Campus and Hospital Clínico de Valencia respectively, and which have extensive experience in the implementation and development of non-commercial clinical trials.
Arrangements will be made for the contracting of the insurance policy.

Development phase (18 months):
The respective UICECs will assign the monitor in charge of the project management and monitoring tasks, who will accompany the research team throughout the clinical trial and will document its development, in accordance with the good clinical practice guidelines and the Monitoring Plan established for this study.
The Hospital de Bellvitge team will be in charge of the geriatric assessment carried out by the local investigators.
- Recruitment: It will be carried out by the local investigators of the participating hospitals. Patients admitted for acute myocardial infarction ≥70 years will be reviewed daily.
Patients who meet all the inclusion criteria and none of the exclusion criteria will be offered participation in the study. If they accept, they will be randomized to invasive or noninvasive strategy. Clinical cardiologists and a nursing team in each hospital will participate in this phase. The estimated duration of this phase is 1.5 years.
- In-hospital geriatric assessment: This will be performed by the local investigators, duly advised by the Hospital de Bellvitge team (Dr Albert Ariza-Solé, Dr Francesc Formiga).
- Follow-up: Patients will be followed up for one year after the index hospitalization by the local investigators. Geriatric assessment during follow-up will also be carried out by the local investigators, duly advised by the Hospital de Bellvitge team.
- Data collection: Data collection will be carried out systematically and continuously throughout the recruitment and follow-up phase by the local investigators of the participating hospitals. The data will be available in the specifically created database.

3. Analysis of results (2 months): Once the follow-up has been completed, the results will be analyzed by an external statistical consultant.

4. Preparation of manuscript and publication of results (2 months): The final report and manuscript are expected to be completed during the last year of the project. This activity will be the responsibility of the PIs of the project (A. Ariza-Solé and G. Miñana) and the advisory team (H. Bueno).

The complete list of research team members is specified on page 18.

Expediente Nº

INVESTIGADOR/A PRINCIPAL: Gema Miñana Escrivá

INTENSIFICACIÓN

En caso de solicitar Intensificación, describir la actividad asistencial (consulta, actividad quirúrgica, exploraciones complementarias, actividad en servicios centrales, etc.) que justifique la solicitud, en relación con la actividad de investigación desarrollada por el Investigador Principal (IP) (Ajústese al espacio disponible)

Expediente Nº

INVESTIGADOR/A PRINCIPAL: Gema Miñana Escrivá

MEMORIA DE SOLICITUD PROYECTO DE INVESTIGACIÓN EN SALUD
SECCIÓN PLAN DE TRABAJO
(Ajústese al espacio disponible. Puede incorporar hasta un máximo de 8 líneas de Actividad/Tarea)

CRONOGRAMA

ACTIVIDAD / TAREA	PERSONA/S INVOLUCRADAS		E	F	M	A	M	J	J	A	S	O	N	D
Protocol design, database development and team building (2 months)	A. Ariza, G. Miñana, H. Bueno	1º Año	✓	✓										
		2º Año												
		3º Año												

ACTIVIDAD / TAREA	PERSONA/S INVOLUCRADAS		E	F	M	A	M	J	J	A	S	O	N	D
Recruitment. Conservative vs. invasive management. Geriatric assessment. Prospective data inclusion (18 months).	Investigadores locales	1º Año			✓	✓	✓	✓	✓	✓	✓	✓	✓	✓
		2º Año	✓	✓	✓	✓	✓	✓	✓	✓				
		3º Año												

ACTIVIDAD / TAREA	PERSONA/S INVOLUCRADAS		E	F	M	A	M	J	J	A	S	O	N	D
Clinical follow-up. Geriatric assessment at one year. Inclusion of information in the electronic CRF (18 months).	Investigadores locales	1º Año												
		2º Año			✓	✓	✓	✓	✓	✓	✓	✓	✓	✓
		3º Año	✓	✓	✓	✓	✓	✓	✓	✓				

ACTIVIDAD / TAREA	PERSONA/S INVOLUCRADAS		E	F	M	A	M	J	J	A	S	O	N	D
Data analysis (2 months)	Asesoría estadística externa	1º Año												
		2º Año												
		3º Año									✓	✓		

ACTIVIDAD / TAREA	PERSONA/S INVOLUCRADAS		E	F	M	A	M	J	J	A	S	O	N	D
Preparation of reports, preparation of manuscripts and dissemination of results (2 months)	A. Ariza, G. Miñana, H. Bueno	1º Año												
		2º Año												
		3º Año											✓	✓

Expediente Nº

INVESTIGADOR/A PRINCIPAL: Gema Miñana Escrivá

MEMORIA DE SOLICITUD PROYECTO DE INVESTIGACIÓN EN SALUD
SECCIÓN PLAN DE TRABAJO

Inserte (si lo desea) una imagen con un cronograma. **Máximo un fichero de imagen formato jpg**

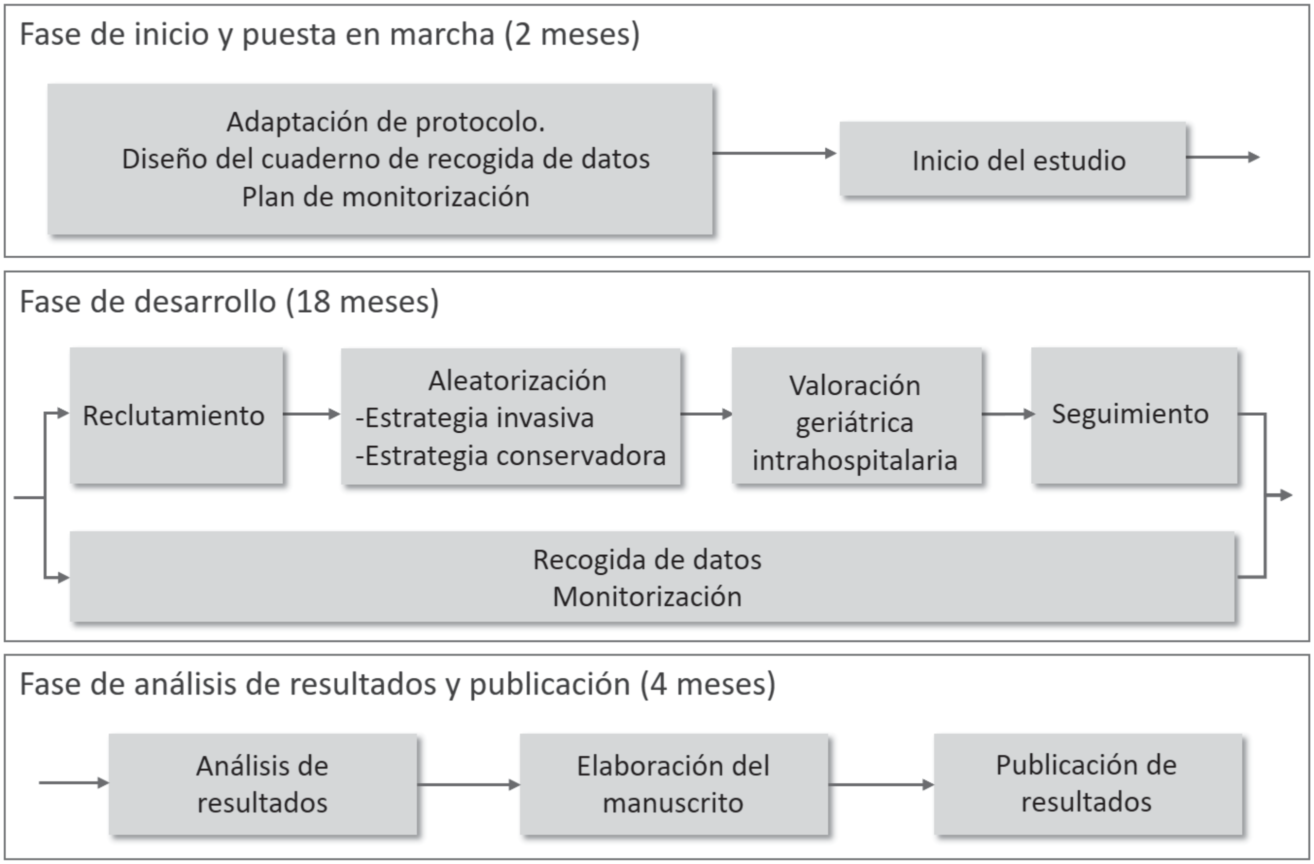

Expediente Nº	
INVESTIGADOR/A PRINCIPAL: Gema Miñana Escrivá	

MEMORIA DE SOLICITUD PROYECTO DE INVESTIGACIÓN EN SALUD
SECCIÓN EXPERIENCIA DEL EQUIPO

Experiencia del equipo investigador sobre el tema. **(Ajústese al espacio disponible)**

The present research project has been designed within the framework of the scientific activities of the Spanish Society of Cardiology and specifically of its Geriatric Cardiology Section to provide a coordinated response at the national level to a major public health problem. The participating researchers all belong to groups with extensive experience and a proven reputation in the study of elderly patients with comorbidities and ACS in Spain. The co-IP of the project at the Hospital de Bellvitge, Dr Albert Ariza-Solé, is currently president of the Geriatric Cardiology Section of the Spanish Society of Cardiology, and is coordinator of several active national registries on comprehensive geriatric assessment in elderly patients with ACS.
A balanced composition of clinical and interventional cardiologists has been achieved, so that the protocol is endorsed by the Sections of Hemodynamics, Ischemic Cardiopathy and Geriatric Cardiology of the Spanish Society of Cardiology. The most relevant and active centers in research on ACS, advanced age, frailty and comorbidities are represented in the research team.

The present project is in the context of the research line of the Hospital Clínico de Valencia on the frail elderly with ACS, led by Dr. Juan Sanchis, which will be developed within the network of CIBER CV (CB16/11/00420A; PI: Juan Sanchis). Recent contributions to this line are briefly detailed below:
-FIS grant PI 11/01595. "Randomized comparison between invasive and conservative strategies in patients with non-ST-segment elevation acute coronary syndrome and comorbidities". PI: Dr. Juan Sanchis
-FIS grant 15/00837. "Randomized comparison between a frailty intervention strategy versus the usual strategy in frail patients after acute myocardial infarction". PI: Dr. Juan Sanchis
-Grant RTC-2016-4684-1. "Development of a new technique Evaluation of intramuscular adipose tissue by dual energy densitometry". Several INCLIVA Research Groups participate, among them the Clinical Cardiology Group led by Dr. Juan Sanchis. Years 2017-2019.
-Juan Sanchis, Vicente Ruiz, Clara Bonanad, Ernesto Valero, Maria Arantzazu Ruescas-Nicolau, Yasmin Ezzatvar, Clara Sastre, Sergio García-Blas, Anna Mollar, Vicente Bertomeu-González, Gema Miñana, and Julio Núñez. Prognostic value of geriatric conditions beyond age after acute coronary syndrome. Mayo Clin Proceed 2017 (in press).
-Veronese N, Cereda E, Stubbs B, Solmi M, Luchini C, Manzato E, Sergi G, Manu P, Harris T, Fontana L, Strandberg T, Amieva H, Dumurgier J, Elbaz A, Tzourio C, Eicholzer M, Rohrmann S, Moretti C, D'Ascenzo F, Quadri G, Polidoro A, Lourenço RA, Moreira VG, Sanchis J, Scotti V, Maggi S, Correll CU. Risk of cardiovascular disease morbidity and mortality in frail and pre-frail older adults: results from a meta-analysis and exploratory meta-regression analysis. Ageing Res Rev 2017;35:63-73.
-Alegre O, Ariza-Solé A, Vidán MT, Formiga F, Martínez-Sellés M, Bueno H, Sanchís J, et al.Impact of Frailty and Other Geriatric Syndromes on Clinical Management and Outcomes in Elderly Patients With Non-ST-Segment Elevation Acute Coronary Syndromes: Rationale and Design of the LONGEVO-SCA Registry. Clin Cardiol 2016;39:373-7.
-Nuñez J, Ruiz V, Bonanad Cl, Miñana G, García-Blas S, Valero E, Nuñez E, Sanchis J. Percutaneous Coronary Intervention and Recurrent Hospitalizations in Elderly Patients with Non ST-segment Acute Coronary Syndrome: The Role of Frailty. Int J Cardiol 2017;228:456 -458.
-Sanchis J, Núñez E,Barrabés JA, et al. Randomized comparison between the invasive and conservative strategies in comorbid elderly patients with non-ST elevation myocardial infarction. Eur J Intern Med 2016;35:89-94
-Juan Sanchis, Eduardo Núñez, Vicente Ruiz, et al. Usefulness of clinical data and biomarkers for the identification of frailty after acute coronary syndromes. Can J Cardiol 2015; 31:1462-8.
-Sanchis J, Bonanad Cl, Ruiz V, et al. Frailty and other geriatric conditions for risk stratification of older patients with acute coronary syndrome. Am Heart J 2014;168:784-791.
-Patricia Palau, Julio Núñez, Juan Sanchis, Oliver Husser, Vicente Bodí, Eduardo Núñez, Gema Miñana, et al. Differential Prognostic Effect of Revascularization According to a Simple Comorbidity Index in High-Risk non ST-Segment Elevation Acute Coronary Syndromes. Clin Cardiol 2012;35(4):237-43.
-Sanchis J, Núñez J, Bodí V, et al. Influence of comorbidities on one-year outcomes in non-ST-segment elevation acute coronary syndrome, Mayo Clinic Proceed 2011;86:291-296.

Expediente Nº

INVESTIGADOR/A PRINCIPAL: Gema Miñana Escrivá

MEMORIA DE SOLICITUD PROYECTO DE INVESTIGACIÓN EN SALUD
SECCIÓN MARCO ESTRATÉGICO

(Ajústese al espacio disponible)

1. Capacidad del proyecto de abordar los objetivos y prioridades enmarcadas en el reto Salud, Cambio Demográfico y Bienestar de la Estrategia Española de Ciencia y Tecnología y de Innovación.
2. Relevancia de la propuesta a la investigación clínico-traslacional.

1.The project fits within the thematic priorities and priority lines of research included in the present call and in the H2020 project ("Aging and frailty", as well as prevention of dependence). The number of patients admitted to our hospitals with acute myocardial infarction, advanced age and frailty is increasing. This population has a high frequency of readmissions and adverse events after discharge, with an evident impact on the health care system. The most appropriate management of these patients after infarction is not well understood. Clinical practice guidelines are essentially based on the results of clinical trials, which generally do not include these patients. Moreover, paradoxically, these patients, who are at greater risk, tend to be treated worse, under the assumption that it is difficult to interfere with this poor prognosis. The results of the project will provide information of undoubted clinical and care impact by evaluating the possible benefit of a specific intervention strategy in frail patients, which will guide the clinical cardiologist's decision making in this population that is so frequent in our hospitals. The fact that the project is multicenter, including a significant number of Spanish tertiary hospitals, and that it is framed within the priority activities and objectives of the Spanish Society of Cardiology and its Geriatric Cardiology Section, will provide great amplification of its results and the corresponding social impact. In addition, an important bibliometric impact is expected given that there are no publications with the design proposed by the project.

2.The relevance of the proposal to clinical-translational research is based on:
(a) investigating the best therapeutic strategy for the frail elderly with acute coronary syndrome, which is increasingly frequent in our hospitals.
b) collaborative synergies between the essentially clinical approach to the problem and the aspect related to coronary anatomy and percutaneous revascularization.
c) synergies of collaboration between centers given the multicenter nature of the study.
d) collaborative synergies between the geriatrics and cardiology forums.
e) collaborative synergies within CIBER-CV.

Expediente Nº	

INVESTIGADOR/A PRINCIPAL: Gema Miñana Escrivá

MEMORIA DE SOLICITUD PROYECTO DE INVESTIGACIÓN EN SALUD
SECCIÓN MEDIOS DISPONIBLES

(Ajústese al espacio disponible)

Medios disponibles para la realización del proyecto.

-Guarantee in the inclusion and collection of 10-15 patients by each of the participating hospitals:

These hospitals are tertiary hospitals with a high level and complexity of care. All of them have Hemodynamic Units, Hospitalization Room and Coronary Unit, as well as Outpatient Clinics for patient follow-up. Each of these centers guarantees a balanced team of clinical and interventional cardiologists with specialized nursing support.

- Geriatric advisory support from the HUB Geriatrics Program.

-Facilities corresponding to the respective University Campuses and Research Institutes, IDIBELL and INCLIVA, with recognition of excellence by the ISCIII.

-Database platform of the IMIM of Barcelona.

The following is the list of the list of Investigators of the different centers [Principal Investigator (PI), Collaborating Investigator (CI) and other collaborators (OC: collaborators who have confirmed their participation in the study by signing a letter of acceptance of the commitment to participate included in Annex 2, but who due to incompatibility problems will not do so as FIS investigators)]:

-Hospital Clínico de Valencia: Juan Sanchís (OC), Gema Miñana (PI), Paolo Racugno (IC), Martina Amiguet (IC).

-Hospital Universitari de Bellvitge-IDIBELL: Albert Ariza Solé (PI), Joan Antoni Gómez-Hospital (CO), Francesc Formiga (IC), Oriol Alegre (IC).

-Germans Trias i Pujol University Hospital (Badalona): Cinta Llibre (IC), Eduard Fernández (PO).

-Vall d'Hebron Hospital: José Barrabés (CO), Bruno García del Blanco (CO), Aleix Olivella (IC).

-Sant Pau Hospital: Miquel Vives (IC), Alex Sionis (OC).

-Moisès Broggi Hospital (Sant Joan Despí, Barcelona): Antoni Carol (IC).

-Gregorio Marañón General University Hospital (Madrid): Iago Sousa (IC), Manuel Martínez-Sellés (OC), Jaime Elizaga (OC).

-Arganda Hospital: Adolfo Villa (CO).

-Ramón y Cajal Hospital: Marcelo Sanmartín (CO), Rosana Hernández (CO), Luisa Salido (IC), Álvaro Marco (IC).

Expediente Nº

INVESTIGADOR/A PRINCIPAL: Gema Miñana Escrivá

MEMORIA DE SOLICITUD PROYECTO DE INVESTIGACIÓN EN SALUD
SECCIÓN JUSTIFICACIÓN DETALLADA DE LAS PARTIDAS PRESUPUESTARIOS SOLICITADAS
(Ajústese al espacio disponible)

This budget estimate is part of the overall project, which also includes the request from the Hospital de Bellvitge team (PI Dr Albert Ariza-Solé). This report includes a budget for the elaboration and maintenance of the electronic CRF, intensification of the PI, contracting of an external statistical consultant, as well as an additional budget for publications and travel and per diems. The overall budgeted amount included in this request amounts to 83500 euros, which should be added to the amount requested below:

-Contracting the insurance policy: €18000.
-Monitoring of the study (INCLIVA): 25,000 euros.
-Travel for meetings and congresses:

During the first and second year, about 2 annual meetings will be required to advise on geriatric assessment and to know the development of the study, and its main incidences. In the last months of the project, trips are planned to plan the analyses and to attend national and international congresses in the field of cardiology and geriatrics to disseminate the results of the project. A budget of 3000 euros is budgeted.

-Publications: The publication of results in high impact national and international journals, both in the field of cardiology and geriatrics, is planned. In order to favor the dissemination of the findings of the project, we plan to prepare publications in open access format. A budget of 2500 euros is budgeted.

Expediente Nº	INVESTIGADOR/A PRINCIPAL:<br>Gema Miñana Escrivá

MEMORIA DE SOLICITUD PROYECTO DE INVESTIGACIÓN EN SALUD
SECCIÓN PRESUPUESTO

Presupuesto solicitado

¿Solicita Intensificación del Investigador Principal (IP)? ○ SI ◉ NO

La solicitud de Intensificación es incompatible con la solicitud de financiación para otro tipo de personal a cargo del proyecto **Euros**

1. Gastos de Personal o Intensificado

Los costes de contratación imputables a la subvenciónse ajustarán a las tablas salariales dictadas al efecto.

**Subtotal Gastos de Personal :**	

2. Gastos de Ejecución

A) Adquisición de bienes y contratación de servicios

(Bienes inventariables, material fungible y gastos complementarios)

Contratación de la póliza de seguro	18.000
Monitorización del estudio	25.000
Publicaciones	2.500
**Subtotal Gastos Bienes y Servicios :**	**45.500**

B) Gastos de Viajes

Viajes para reuniones y congresos	3.000
**Subtotal Gastos Viajes :**	**3.000**
**Subtotal Gastos Ejecución :**	**48.500**
**Total Solicitado :**	**48.500**
**Total + 21% Costes Indirectos :**	**58.685**

Expediente Nº	
INVESTIGADOR/A PRINCIPAL: Gema Miñana Escrivá	

MEMORIA DE SOLICITUD PROYECTO DE INVESTIGACIÓN EN SALUD
SECCIÓN ANEXOS

INTRODUZCA TEXTO COMO ANEXO **Máximo 3 páginas (15.700 caracteres)**

PATIENT INFORMATION CONSENT

You are offered the possibility of participating in the research project entitled "Randomized comparison between invasive and conservative strategies in fragile patients with acute myocardial infarction without ST-segment elevation" which is being carried out in the Cardiology Department and which has already been evaluated and approved by the Clinical Research Ethics Committee of the Hospital Clínico Universitario de Valencia.

Background
Although the benefit of routinely performing cardiac catheterization in patients with an acute myocardial infarction of your characteristics has been demonstrated, it is not known whether this also occurs in the presence of a fragile state such as yours. The questionnaire you have completed suggests that you may have frailty, which is defined by an increased vulnerability to disease in general.

What is the aim of this study?
The aim of this study is to analyze whether routinely performing cardiac catheterization improves the prognosis of acute myocardial infarction of the characteristics you suffer from, in patients who also suffer from frailty.

Why have you been asked to participate?
You have been asked to participate in this study because you have been admitted for an acute myocardial infarction and are frail.

What type of tests or procedures will you be asked to perform?
You will be randomly assigned to have a cardiac catheterization routinely or to have it done only if you have chest pain again or if a serious abnormality is detected if you have a stress test before discharge. Participation in the present project does not imply any alteration in the treatment you are undergoing and any treatment you may be given based on the studies carried out will always be at your doctor's discretion. If you undergo catheterization, the risks of the test will be explained in the corresponding informed consent form.

What are the general risks of participating in this study?
No additional risks to you are anticipated. A separate sheet will explain what a cardiac catheterization consists of.

What are the benefits of participating in this study?
It is quite possible that the results obtained in this research will have little diagnostic or predictive value for you, but it may help to better understand your disease and improve the prognosis and treatment of future patients.

What will happen if I decide not to participate in this study?
Your participation in this study is completely voluntary. If you decide not to participate in the study, this will not change the treatment and follow-up of your disease by your doctor or the other health care personnel who take care of your disease. Likewise, you may withdraw from the study at any time, without having to give explanations.

Who can I ask in case of doubt?
It is important that you discuss with any of the investigators of this project any details or doubts that may arise before signing the consent for your participation. Likewise, you may request any explanation you wish about any aspect of the study and its implications throughout the study by contacting the principal investigator of the project, Dr. Juan SanchisForés, at telephone number 961973807.

Confidentiality:

All your data, as well as all medical information related to your disease will be treated with absolute confidentiality by the staff in charge of the research. Likewise, if the results of the study were susceptible of publication in scientific journals, at no time will personal data of the patients who have collaborated in this research be provided.
As provided for in Organic Law 15/1999 on the Protection of Personal Data, you may exercise your right to access, rectify or cancel your data by contacting the principal investigator of this study.

INFORMED CONSENT

Project Title: Randomized comparison between invasive and conservative strategies in fragile patients with non-ST-segment elevation myocardial infarction.
Principal Investigator: Dr. Gema Miñana
Service: Cardiology

I, __________________________________ have been informed by Dr.__________________, collaborator of the above research project, and declare that:

- I have read the Information Sheet given to me.
- I have been able to ask questions about the study
- I have received satisfactory answers to my questions
- I have received sufficient information about the study

I understand that my participation is voluntary

I understand that all my data will be treated confidentially.

I understand that I can withdraw from the study:
- Whenever I want
- Without having to give explanations
- Without repercussion on my medical care.

I hereby agree to participate in this study,

Signature of patient: Signature of Investigator:
Date: Date:

Attachment 2. Letter of commitment to participate in the project

On the occasion of the FIS application "Randomized comparison between invasive and conservative strategies in frail elderly patients with non-ST-segment elevation myocardial infarction (MOSCA-FRAIL)", the group of the hospital -- (name of the center) hereby confirm our interest and commitment to the project.
Therefore, Dr/s-- hereby confirms its commitment to to collaborate in this study, although for reasons of incompatibility he cannot be formally included as an associated collaborating
formally as a collaborating researcher associated to this project call.

Sincerely
Dr.

Signature and date of the researcher/s

Expediente Nº

INVESTIGADOR/A PRINCIPAL: Gema Miñana Escrivá

INTRODUZCA UNA IMAGEN COMO ANEXO **Máximo un fichero de imagen formato jpg**

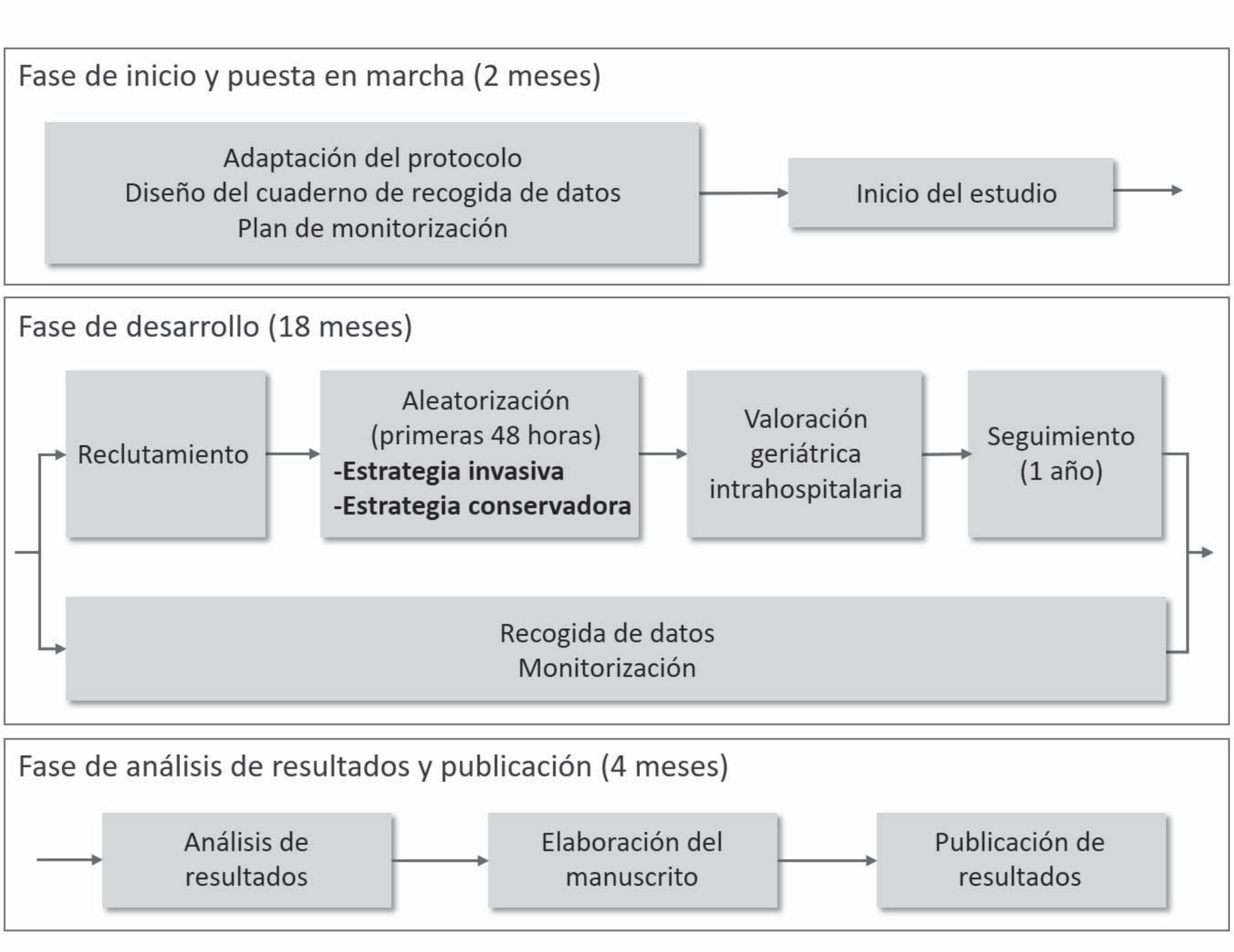

Supplement: Supplement 1. — Trial Protocol [file jamanetwopen-e240809-s001.pdf]
