# Supplementary material for: Invasive Treatment Strategy in Adults With Frailty and Non–ST-Segment Elevation Myocardial Infarction: A Secondary Analysis of a Randomized Clinical Trial
Source: JAMA Netw Open. 2024 Mar 6;7(3):e240809. doi: 10.1001/jamanetworkopen.2024.0809 (PMC10918507; doi:10.1001/jamanetworkopen.2024.0809)
Supplement: Supplement 2. — eTable 1. Baseline Patient Characteristics eTable 2. Causes of Noncardiac Death eTable 3. Effect of the Invasive Treatment on the Rate of Each Recurrent Event While Adjusting the Estimates for Informative Censoring Due to Death as a Terminal Event eFigure 1. CONSORT Flow Diagram eFigure 2. RMST Curve for All-Cause Mortality [file jamanetwopen-e240809-s002.pdf]

Supplementary Online Content

Sanchis J, Bueno H, García-Blas S, et al. Invasive treatment strategy in adults with frailty and non–ST-segment elevation myocardial infarction: a randomized clinical trial. *JAMA Netw Open*. 2024;7(3):e240809.
doi:10.1001/jamanetworkopen.2024.0809

eTable 1. Baseline Patient Characteristics
eTable 2. Causes of Noncardiac Death
eTable 3. Effect of the Invasive Treatment on the Rate of Each Recurrent Event While Adjusting the Estimates for Informative Censoring Due to Death as a Terminal Event
eFigure 1. CONSORT Flow Diagram
eFigure 2. RMST Curve for All-Cause Mortality

This supplementary material has been provided by the authors to give readers additional information about their work.

© 2024 Sanchis J et al. *JAMA Network Open*.

eTable 1. Baseline Patient Characteristics

	Invasive	Conservative	Standardized differences
**N = 167**	**84**	**83**	
**Demographic data**			
Age (years)	86 (5)	85 (5)	.218
Male	32 (38)	47 (57)	.401
Diabetes	50 (60)	43 (52)	.155
Insulin treatment	19 (23)	19 (23)	.006
Hypertension	77 (92)	76 (92)	.004
Hypercholesterolemia	63 (75)	65 (78)	.078
Current smoker	3 (3.6)	2 (2.4)	.230
Peripheral artery disease	9 (11)	9 (11)	.004
Chronic kidney disease	39 (46)	35 (42)	.085
Dialysis	3 (3.6)	2 (2.4)	.068
Prior myocardial infarction	19 (23)	32 (39)	.349
Prior PCI	19 (23)	33 (40)	.374
Prior CABG	5 (6.0)	11 (13)	.248
Prior history of atrial fibrillation	26 (31)	19 (23)	.181
Prior stroke	13 (16)	17 (21)	.203
Prior admission for heart failure	13 (16)	16 (19)	.100
**Hemodynamic data**			
Systolic blood pressure (mmHg)	139 (27)	141 (23)	.060
Diastolic blood pressure (mmHg)	69 (16)	70 (12)	.105
Heart rate (beats/min)	77 (15)	79 (21)	.095
Killip class			.0049
I	67 (80)	61 (74)	
II	11 (13)	19 (23)	
III	6 (7.1)	3 (3.6)	
**ECG**			

© 2024 Sanchis J et al. *JAMA Network Open*.

Normal ECG	26 (31)	27 (33)	.127
ST-segment depression	34 (41)	25 (30)	.187
T-wave inversion	9 (11)	16 (19)	.043
Left bundle branch block	10 (12)	10 (12)	.040
Pacemaker	5 (6.0)	5 (6.0)	.003
**Blood test**			
Haemoglobin (g/dl)	12.4 (1.6)	12.4 (1.8)	.008
Creatinine (mg/dl)	1.4 (0.9)	1.3 (0.7)	.056
**Geriatric conditions**			
Clinical Frailty Scale, n (%)			.073
4	23 (27)	20 (24)	
5	32 (38)	40 (48)	
6	26 (31)	22 (27)	
7	3 (4)	1 (1)	
Frail scale (points)	2.5 (1.2)	2.5 (1.1)	.015
Charlson index (points)	2.5 (1.9)	3.0 (2.2)	.234
Barthel index (points)	75 (23)	75 (20)	.001
Pfeiffer test (errors)	1.8 (2.2)	2.0 (2.2)	.140

Continuous variables are presented as mean (SD), and categorical variables as absolute values (%)

Abbreviations: PCI = Percutaneous coronary intervention, CABG: Coronary artery bypass graft

© 2024 Sanchis J et al. *JAMA Network Open*.

eTable 2. Causes of Noncardiac Death

	Invasive N=84	Conservative N=83	Total
Bleeding	5	0	5
During the first year	4	0	
After the first year	1	0	
Stroke	4	2	6
During the first year	4	1	
After the first year	0	1	
Other neurologic disease	3	1	4
During the first year	1	0	
After the first year	2	1	
Pulmonary disease	4	7	11
During the first year	1	1	
After the first year	3	6	
Gastrointestinal disease	3	2	5
During the first year	2	1	
After the first year	1	1	
Malignancy	4	2	6
During the first year	1	0	
After the first year	3	2	
Diabetes de compensation	0	1	1
During the first year	0	1	
After the first year	0	0	
Multiorgan failure	1	2	3
During the first year	1	0	
After the first year	0	2	
Sepsis	2	2	4
During the first year	0	1	
After the first year	2	1	
Peripheral artery disease	0	1	1
During the first year	0	1	
After the first year	0	0	
Fall	0	1	1
During the first year	0	0	
After the first year	0	1	
Urinary infection	1	1	2
During the first year	0	0	
After the first year	1	1	

© 2024 Sanchis J et al. *JAMA Network Open*.

eTable 3. Effect of the Invasive Treatment on the Rate of Each Recurrent Event While Adjusting the Estimates for Informative Censoring Due to Death as a Terminal Event

	**IRR**	**95% CI**	***P* value**
Reinfarction	1.13	0.61 to 2.10	.69
Revascularization	1.01	0.47 to 2.16	.98
Unstable angina	0.50	0.23 to 1.08	.08
Readmission for heart failure	1.41	0.88 to 2.25	.15
Readmission for other cardiac reasons	0.50	0.23 to 1.08	.08
Stroke	1.30	0.25 to 6.91	.76
Readmission for bleeding	4.09	0.90 to 18.6	.07
Readmission for other noncardiac reasons	0.70	0.41 to 1.19	.19

Abbreviations: IRR = Incidence rate ratio. CI = Confidence interval

© 2024 Sanchis J et al. *JAMA Network Open*.

eFigure 1. CONSORT Flow Diagram

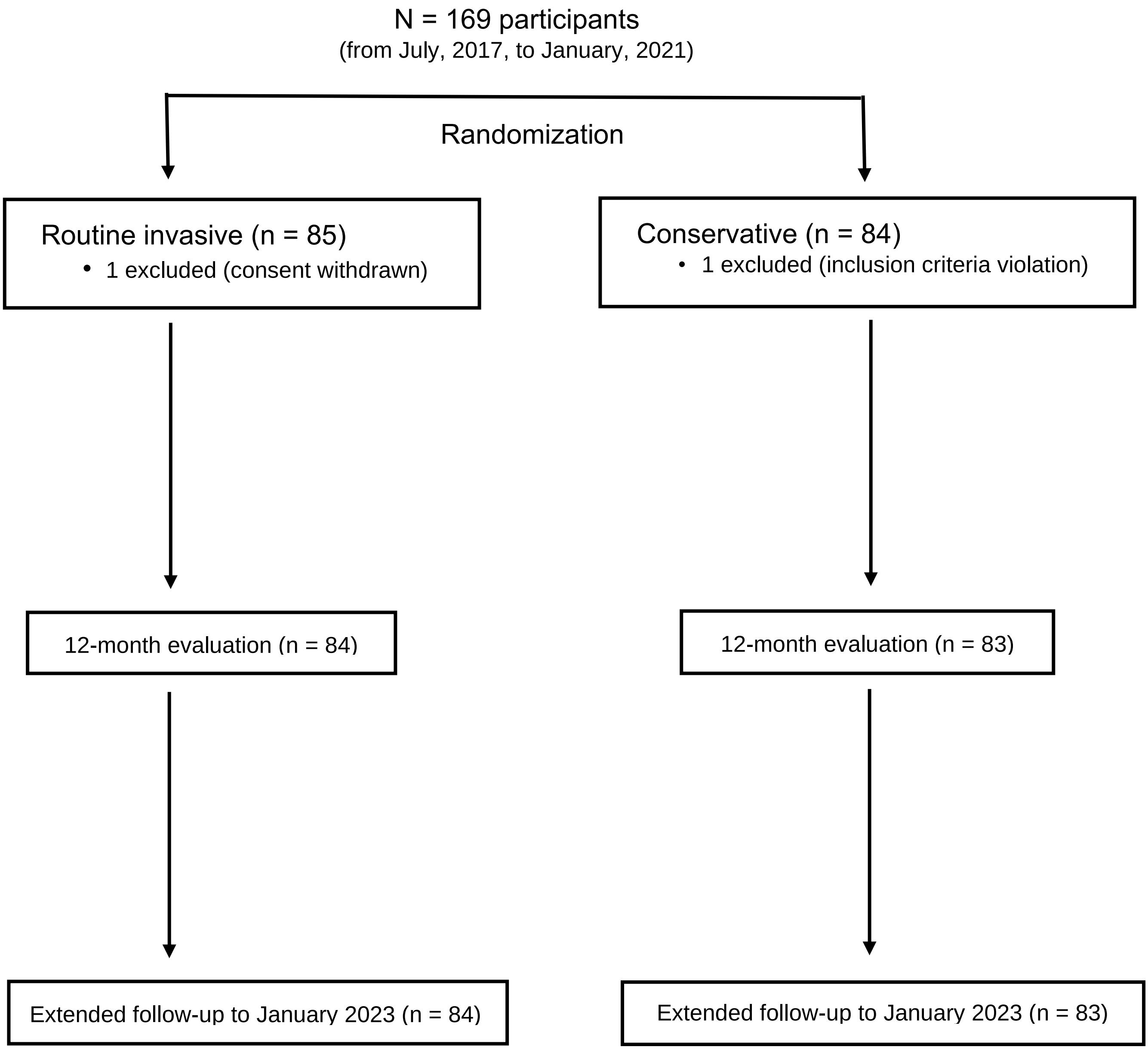

© 2024 Sanchis J et al. *JAMA Network Open*.

eFigure 2. RMST Curve for All-Cause Mortality

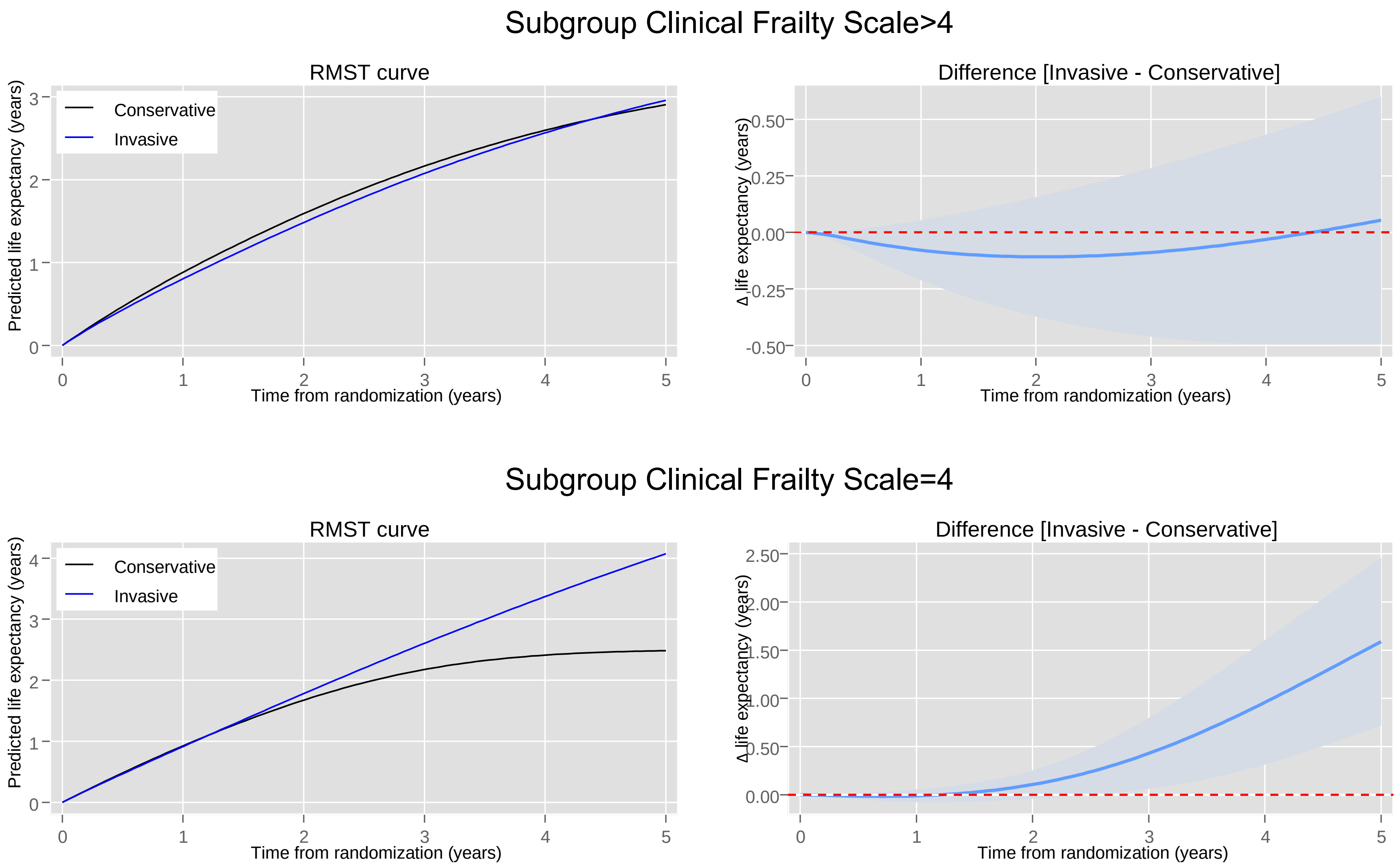

Figure legend: Restricted mean survival curves for all-cause mortality, comparing the conservative and invasive treatment strategies in the subgroups with Clinical Frailty scale >4 (top) and 4 (bottom), using the propensity score model. Abbreviations: RMST = Restricted mean survival time

© 2024 Sanchis J et al. *JAMA Network Open*.
