# Supplementary material for: Invasive Treatment Strategy in Adults With Frailty and Non–ST-Segment Elevation Myocardial Infarction: A Secondary Analysis of a Randomized Clinical Trial
Source: JAMA Netw Open. 2024 Mar 6;7(3):e240809. doi: 10.1001/jamanetworkopen.2024.0809 (PMC10918507; doi:10.1001/jamanetworkopen.2024.0809)
Supplement: Supplement 3. — Data Sharing Statement [file jamanetwopen-e240809-s003.pdf]

Data Sharing Statement

Sanchis. Invasive Treatment Strategy in Adults With Frailty and Non–ST-Segment Elevation Myocardial Infarction. *JAMA Netw Open*. Published March 06, 2024.
doi:10.1001/jamanetworkopen.2024.0809

Data
Data available: Yes
Data types: Deidentified participant data
How to access data: The MOSCA-FRAIL study is committed to responsible data sharing. Data sharing will be considered upon reasonable request. All collaborations will be made after a collaboration agreement
When available: With publication

Supporting Documents
Document types: None

Additional Information
Who can access the data: The MOSCA-FRAIL study is committed to responsible data sharing. Data sharing will be considered upon reasonable request. All collaborations will be made after a collaboration agreement
Types of analyses: The MOSCA-FRAIL study is committed to responsible data sharing. Data sharing will be considered upon reasonable request. All collaborations will be made after a collaboration agreement
Mechanisms of data availability: After approval of a proposal
